# Identification of HYPK-Interacting Proteins Reveals Involvement of HYPK in Regulating Cell Growth, Cell Cycle, Unfolded Protein Response and Cell Death

**DOI:** 10.1371/journal.pone.0051415

**Published:** 2012-12-10

**Authors:** Kamalika Roy Choudhury, Swasti Raychaudhuri, Nitai P. Bhattacharyya

**Affiliations:** Crystallography and Molecular Biology Division, Saha Institute of Nuclear Physics, Kolkata, West Bengal, India; Blaise Pascal University, France

## Abstract

Huntingtin Yeast Two-Hybrid Protein K (HYPK) is an intrinsically unstructured huntingtin (HTT)-interacting protein with chaperone-like activity. To obtain more information about the function(s) of the protein, we identified 27 novel interacting partners of HYPK by pull-down assay coupled with mass spectrometry and, further, 9 proteins were identified by co-localization and co-immunoprecipitation (co-IP) assays. In neuronal cells, (EEF1A1 and HSPA1A), (HTT and LMNB2) and (TP53 and RELA) were identified in complex with HYPK in different experiments. Various Gene Ontology (GO) terms for biological processes, like protein folding (GO: 0006457), response to unfolded protein (GO: 0006986), cell cycle arrest (GO: 0007050), anti-apoptosis (GO: 0006916) and regulation of transcription (GO: 0006355) were significantly enriched with the HYPK-interacting proteins. Cell growth and the ability to refold heat-denatured reporter luciferase were decreased, but cytotoxicity was increased in neuronal cells where *HYPK* was knocked-down using *HYPK* antisense DNA construct. The proportion of cells in different phases of cell cycle was also altered in cells with reduced levels of HYPK. These results show that HYPK is involved in several biological processes, possibly through interaction with its partners.

## Introduction

Huntingtin Yeast Two-Hybrid Protein K (HYPK, gene ID: 25764) was first identified as a Huntingtin (HTT)-interacting protein using a yeast two-hybrid assay [1]. We previously confirmed this interaction by immunoprecipitation (IP) and co-localization in cultured cells [2]. It has been further shown that HYPK belongs to the family of intrinsically unstructured proteins (IUP) with a pre-molten globule like conformation [3]. HYPK exhibits chaperone-like activity *in vitro* and *in vivo* without being homologous to any known chaperone and can reduce formation of aggregates and apoptosis in a cell model of Huntington’s disease (HD) [2]. The catalytic and auxiliary subunits of human N^α^-terminal-acetyltransferase (NatA) complex (NAA10 and NAA15 respectively) that participate in co-translational N-terminal acetylation of proteins, have been found to be associated with HYPK in polysome fraction [4]. Reduced expression of human *NAA10* and *NAA15* resulted in a decrease in the amount of HYPK protein indicating that these components maintain stability of HYPK. In addition, it has also been shown that HYPK is necessary for efficient N-terminal acetylation of known NatA substrate. However, there were no significant effects of HYPK knock-down on the expression of NAA10 and NAA15. Knock-down of *NAA10, NAA15* or *HYPK* increases the aggregates formed by mutant HTT. When the expression of HYPK was reduced, HeLa cells were arrested in G0/G1 phase of cell cycle. This result suggests that HYPK is involved in post-translational modification of proteins and cell cycle regulation [4]. HYPK was co-purified with ribosome-associated MPP11/DNAJC2/Hsp70L1 complex along with ARD1/NAA10 and NAA15 [5]. HYPK thus may act as a binding partner to one or more of these proteins. In addition, 8 proteins (*viz.*, CHD3, GC, MDFI, PSME3, QKI, RBPMS, RHOXF2 and TH1L) were identified to interact with HYPK in yeast two-hybrid assay [6], [7], although none of them were further validated as a binding partner of HYPK.

IUPs are commonly identified in the hubs of protein-protein interaction networks and are able to form complexes with other proteins [8], [9], [10]. Swiss-Prot annotated cellular processes like cell differentiation, transcription, cell cycle, mitosis, meiosis, apoptosis, ubiquitin proteasomal degradation, growth regulation, ribosome biogenesis and mRNA processing are enriched with IUPs [11], [12]. Recently, we have shown that IUPs are significantly over-represented among the proteins involved in HD, Parkinson’s disease (PD) and Alzheimer’s disease (AD). IUPs were reported to be prevalent among the hub proteins (having more than 10 interacting partners) involved in HD pathology as compared to the end proteins. Various biological processes such as neuronal activities, cell proliferation and differentiation, cell structure and motility, apoptosis and its regulation, carbohydrate and protein metabolism, cell cycle, intracellular protein traffic, electron transport and protein targeting and localization are reported to be enriched with IUPs involved in neurodegenerative diseases [13].

Based on the above observations, we hypothesized that HYPK might interact with various other proteins. In this study, we identified 36 novel HYPK-interacting proteins. Our experimental results and bioinformatics analyses confirmed participation of HYPK and its interacting partners in cell growth, cell cycle regulation, maintenance of cell survival and unfolded protein response.

## Materials and Methods

The list of antibodies used and their sources are shown in Table S1.

### Cell Culture, Preparation of DNA Constructs and Transfection

ST*Hdh^Q7^*/*Hdh^Q7^* and ST*Hdh^Q111^*/*Hdh^Q111^* cell lines, obtained as generous gift from Dr. Marcy E. MacDonald, were cultured as published earlier [14], [15]. Neuro2A and HeLa cell lines were procured from National Cell Science Centre (Pune, India) and the growth conditions were similar to those published earlier [16]. Mouse striatal cell lines expressing full-length HTT protein with either 7 glutamine (Q) residues (designated as ST*Hdh^Q7^*/*Hdh^Q7^*) or 111 Q residues (designated as ST*Hdh^Q111^*/*Hdh^Q111^*) represent the normal and diseased condition (i.e., HD) respectively. Gene cloning and transfection were performed as described earlier [2]. Detailed information of human genes cloned in fluorescent expression vectors using PCR primers is shown in Table S2A. A list of all the cloned genes used in this study and their sources is shown in Table S2B. The cloned genes are designated by gene symbols followed by the tags (e.g. HYPK-GFP). Co-localization of proteins was determined using confocal microscopy following methods described earlier [2]. Extent of co-localization between the pixels of the two channels was determined by the square of the Pearson correlation coefficient value (R_p_) generated by in-built software. This squared value, R^2^, is defined as the percentage of variance of the pixels in first channel which is dependent on the variance of that of the second channel.

### Preparation of Soluble Cell Lysate (SCL) from Cultured Mammalian Cell Lines

Cells in culture were washed and scraped with 1 ml of ice-cold phosphate buffered saline (PBS) and collected by centrifugation at 4500 rpm for 3 min at 4°C. The pellets were re-suspended in 50 µl of ice-cold lysis buffer (50 mM Tris, pH 8.0 containing 150 mM sodium chloride, 1.0% NP-40 and 1X protease inhibitor cocktail) followed by 30 min of constant agitation at 4°C. These crude extracts were again centrifuged at 12000 rpm for 20 min at 4°C and the supernatant was collected. The protein content in soluble cell lysate (SCL) was estimated by Bradford assay.

### Pull-down with Purified 6HN-HYPK

Recombinant 6HN-HYPK was purified following the methods described earlier [3]. Pull-down experiments were carried out using ProFound™ Pull-Down PolyHis Protein: Protein Interaction Kit (Thermo Scientific Product no. 21277) as per manufacturer’s instructions. In brief, 200 µg purified 6HN-HYPK (bait) was allowed to bind to Ni-NTA column for 4–5 h at 4°C in a rotating wheel. After thorough washing to remove any unbound bait protein, SCL from HeLa, Neuro2A, ST*Hdh^Q7^*/*Hdh^Q7^* or ST*Hdh^Q111^*/*Hdh^Q111^* cells (∼800 µg) was used as prey and incubated with Ni-NTA column-bound HYPK overnight. In control experiments, only SCL was passed through Ni-NTA column without bound HYPK. The column was then washed thrice with binding buffer (the cell lysis buffer mentioned above) followed by elution of the bound complexes with 300 mM imidazole.

### One-dimensional (1D) and Two-dimensional (2D) SDS-PAGE

Protein complexes obtained using the methods described above were resolved either on 1D SDS-polyacrylamide gradient (4%–16%) gels following standard protocol, or 2D SDS-polyacrylamide gels (12%) following methods described by Bhattacharya et al. [17]. Resolved proteins were visualized after staining with Coomassie Brilliant Blue (CBB). For 2D SDS-PAGE, linear immobilized pH gradient (IPG) strips (7 cm, pH 3–10, Biorad, USA) were rehydrated with the pull-down complexes and electrophoresis was performed as described [17].

### Mass Spectrometry (MS)

The visible bands (in case of 1D gel) or spots (in case of 2D gel) detected in the experimental and control (without the bait HYPK) gels were compared after staining and the unique bands or spots were cut, destained and prepared for mass spectrometry (MS) using the protocols described by Bhattacharya et al. [17]. In brief, the bands/spots were analyzed using a MALDI-TOF/TOF mass spectrometer (4700 Proteomic Analyzer, Applied Biosystems). The MS/MS data were pre-processed by the GPS Explorer™ software version 3.6 (Applied Biosystems) and proteins were identified using the MASCOT search engine, which incorporates a probability based implementation of the MOWSE (**Mo**lecular **W**eight **SE**arch) algorithm for protein annotation using a significance level of ≤0.05.

### Immunocytochemistry for Detection of RelA (p65)

Neuro2A cells grown on coverslips were fixed with 4% paraformaldehyde in PBS for 30 min at 37°C. The fixed cells were then permeabilized using 0.25% (v/v) Triton X-100 in PBS, blocked by 2% BSA (w/v) in PBS and were incubated with anti-mouse p65 and anti-rabbit HYPK antibodies overnight at 4°C. After thorough washing with PBS, cells were incubated with anti-mouse-TRiTC and anti-rabbit-FITC secondary antibodies for 2 h at 37°C and co-localization was analyzed using LSM 510 confocal microscope (Carl Zeiss, Germany).

### Co-immunoprecipitation (co-IP) Studies

The methods used for co-IP were similar to which has been published [15]. Anti-HYPK antibody was used to immunoprecipitate the HYPK-complex. The immunoprecipitated protein complex was eluted using 100 mM glycine, pH 3.0 (referred to as “immunoprecipitate elute” henceforth) and was analyzed by native and SDS-PAGE western blotting.

### Native PAGE-western Transfer

In native PAGE, SDS was added neither in the gel-running buffer nor in sample-loading buffer (each 20 ml 2X native PAGE loading buffer contain 12.5 ml of 0.5 M Tris-Cl, pH 6.8, 2 ml of glycerol, 40 µl of 5% Bromophenol Blue and 5.5 ml of H_2_O). For efficient transfer of the large protein complexes to PVDF membrane, the gel was immersed in 1X SDS-PAGE running buffer and incubated at 75–80°C for 30 min in a hybridization oven (Amersham Life Sciences) followed by transfer and detection using antibodies as described above.

### Antisense Mouse Hypk Cloning to Knock-down HYPK Expression in Mouse Neuronal Cells

To knock-down the expression of HYPK in mouse cells in culture, pRNA-U61/Hygro vector (Genscript, USA), commonly used for cloning siRNA, was utilized. The exon1 of *Mus musculus HYPK* gene (accession no. NM_026318.3) was used to obtain its reverse complementary sequence from http://arep.med.harvard.edu/labgc/adnan/projects/Utilities/revcomp.html. Primers were designed from the reverse complementary strand; position bp 9 to 65 (minus strand). Primer sequence for cloning antisense HYPK is provided in Table S2A. *BamHI* adaptor site in the forward primer and *HindIII* adaptor site in the reverse primer were used. In front of the *HindIII* adaptor sequence in the reverse primer, a poly (T) sequence was fused to ensure the termination of RNA expression from this DNA insert. This antisense-HYPK pU61 clone (designated throughout the manuscript as HYPK-U61) was transfected in Neuro2A and ST*Hdh^Q7^/Hdh^Q7^*cell lines. These transfected cells were then selected with Hygromycin (Invitrogen, USA; final concentration 400 µg/ml) to make stable HYPK knocked-down cell lines. To nullify the effect of the vector, Neuro2A and ST*Hdh^Q7^/Hdh^Q7^*cell lines were transfected with pU61 vectors and stable cell lines were established by selection with Hygromycin as described above.

### Cell Growth

The growth curves for Neuro2A and ST*Hdh^Q7^/Hdh^Q7^* cells and HYPK downregulated Neuro2A and ST*Hdh^Q7^/Hdh^Q7^* cells were compared. To obtain the growth curves, ∼10^5^ cells were seeded in 60 mm petridishes in duplicate. Cell numbers were counted using a haemocytometer (Rohem, India) at 24 h intervals.

### BrdU Cell Proliferation Assay

The proliferative capacity of ST*Hdh^Q7^*/*Hdh^Q7^* cells with endogenous and reduced HYPK expression was determined using BrdU cell proliferation assay kit (Calbiochem, Cat. No. QIA58) following manufacturer’s instructions. In brief, ∼5×10^4^ cells were seeded on 24-well culture plates in triplicate. Cells were transfected with either empty DsRed vector or HYPK-DsRed, and BrdU labels were added 24 h post-transfection. After 12 h of BrdU incorporation, cells were fixed and sequentially incubated with anti-BrdU primary and secondary antibodies. The quantity of BrdU incorporation was estimated by measuring the absorbance at dual wavelengths of 450–540 nm using an Epoch microplate reader (BioTek Instruments, USA).

### Cell Survival (MTT Assay) in Presence and Absence of HYPK

MTT assay was performed using the methods described earlier [2]. In brief, after addition of MTT, ST*Hdh^Q7^*/*Hdh^Q7^* cells were re-incubated in a CO_2_ incubator for 4 h at 33°C. Cells were then dissolved in 0.1N HCl-absolute isopropanol solvent. The colour generated was measured using SmartSpec Plus spectrophotometer (BioRad, USA) taking absorbance at 570 nm with background subtraction done at 630 nm.

### Counting the Aggregates Formed by Mutant HTT

83Q-DsRed (100 ng and 200 ng) was transfected in ST*Hdh^Q7^*/*Hdh^Q7^* cells and HYPK downregulated ST*Hdh^Q7^*/*Hdh^Q7^* cells. The number of cells with and without aggregates were then counted and percentage of cells containing aggregates was determined as described earlier [2].

### Flow Cytometry

Empty vector (GFP) and HYPK-GFP transfected ST*Hdh^Q7^*/*Hdh^Q7^* cells and HYPK downregulated ST*Hdh^Q^* ^7^/*Hdh^Q7^* cells growing in cultures were washed, trypsinized and harvested in PBS and fixed in 70% ethanol overnight at −20°C. After treatment with 1 mg/ml RNase A (Sigma, USA) at 37°C for 60 min, DNA staining was performed using 7-aminoactinomycin D (7-AAD) viability staining solution (BioLegend, USA) at 37°C for 15 min in dark, prior to flow cytometric analysis. Analysis was performed on a FACS calibur flow cytometer (Becton Dickinson, USA). For each sample, 25,000 events were collected and aggregated cells were gated out. Percentage of cells existing within the different phases of the cell cycle was determined using CellQuest Pro software.

### Response to Unfolded Reporter Luciferase Activity after Heat Shock

Luciferase assays were performed using the Tet-Off Advanced Inducible Gene Expression System (BD Biosciences, USA) as described earlier [2]. HYPK downregulated Neuro2A or control (Neuro2A-U61) cells were transfected with pTet-Off-Advanced vector (BD Biosciences) and stable cell lines were obtained by selecting with G418 (Sigma) at a final concentration of 200 µg/ml. These pTet-Off+ve stable cells (designated as Tet^+^) were further transfected with the luciferase expression construct, pTRE-Tight-Luc (BD Biosciences). These stable cells are designated as Tet^+^Luc+ve henceforth. To identify the role of HYPK and its interacting partners in refolding of the heat-denatured reporter luciferase, we overexpressed either DsRed (vector control), HYPK-DsRed, EEF1A1-DsRed, CALM1-DsRed, LMNB2-DsRed or HSPA8-DsRed in the parental Tet^+^Luc+ve Neuro2A-U61 cells and Tet^+^Luc+ve Neuro2A-HYPK U61 cells. After 48 h of transfection cell extracts were prepared from these cells before heat shock (designated as No HS), immediately after heat shock at 43°C for 1 h (designated as HS) and after 6 h of recovery at 37°C following heat shock (designated as HS+R). Relative luciferase activities (in RLU) of these cell extracts at different time points were measured in a Sirius Luminometer (Berthold Detection Systems GmbH, Germany) with the Luciferase Assay System (Promega, USA), according to the manufacturer’s instructions as published earlier [2].

### Bioinformatics Analyses

Several online facilities are available for analysis of various Gene Ontology (GO) terms enriched with a set of genes. In our experience, we find that GeneCodis2 is not only user friendly but it can also be used for enrichment of GO terms for molecular functions, biological processes, cellular components and the Kyoto Encyclopedia of Genes and Genomes (KEGG) pathway. The proportion of proteins in a particular GO term from the input query list is computed and compared with those of proteins coded by human genome as catalogued in the database. The hypergeometric p value was computed after correction for multiple testing [18], [19]. Analysis of HYPK-interacting proteins was performed using GeneCodis2 (http://genecodis.dacya.ucm.es/analysis/). Biological pathway analysis of HYPK-interacting partners for enrichment was performed using GeneCodis2 and Reactome (http://www.reactome.org) databases [20].

### Subcellular Localization and Expressions of HYPK-interacting Proteins

Information of subcellular localizations of the HYPK-interacting proteins was obtained from the online resource Nextprot (http://www.nextprot.org) [21]. Similarly information about expression of genes coding for HYPK-interacting proteins was obtained using Nextprot, BioMart (http://www.ensembl.org/biomart/martview/) and Tissue-specific Gene Expression and Regulation (TiGER) (http://bioinfo.wilmer.jhu.edu/tiger/) databases. In Nextprot, the reported expression of proteins in brain was derived from immunohistochemistry data. BioMart provides information of gene expressions from microarray data described in various external resources and different anatomical regions. In TiGER, the expression of genes in different organs is catalogued based on the numbers of expressed sequence tag (EST).

### Statistical Analysis and Artwork Preparation

All experiments were repeated at least thrice unless otherwise mentioned and mean values, standard deviations and p values for Student’s unpaired two-tailed t test were calculated. For statistical calculations, Microsoft Excel and the online GraphPadQuickCalcs software were used. All the artworks were prepared using Adobe Photoshop CS2.

## Results

### Identification of Novel HYPK-interacting Proteins by Pull-down Followed by Mass Spectrometry (MS)

**Figure 1:**
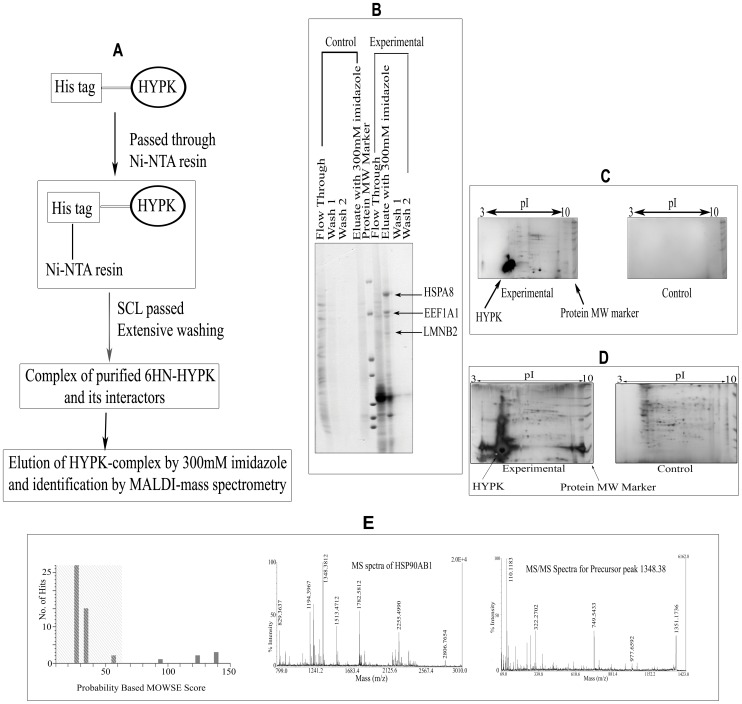
Identification of HYPK-interacting partners using pull-down coupled with MALDI-mass spectrometry. Flow chart for pull-down experiment using purified 6HN-HYPK as bait (***A***); separation of pulled-down proteins by 1D SDS-PAGE using HeLa SCL as prey (***B***); 2D SDS-PAGE using ST*Hdh^Q111^*/*Hdh^Q111^* SCL as prey (***C***), 2D SDS-PAGE with *S*T*Hdh^Q7^*/*Hdh^Q7^* SCL as prey (***D***); probability based MOWSE score and parent MS and subsequent MS/MS spectra for HSP90AB1 (***E***). From the MOWSE score distribution, it is evident that the peptide mass fingerprint search identifying HSP90AB1 (MOWSE score 139) was significant (p≤0.05). MALDI spectra for 5 other proteins and details of MALDI-mass analyses for the identification of HYPK-interacting proteins are provided as Figures S1, S2, S3, S4, and S5 and Table S3 respectively.

The scheme of a typical pull-down experiment is shown in Figure 1, panel A. Pictures of typical 1D and 2D gels are shown in Figure 1, panels B, C and D. All gels were stained with CBB. Representative mass spectrum obtained after trypsin digestion of a band/spot and database search for the protein HSP90AB1 is shown in Figure 1, panel E. Representative mass spectra for 5 other proteins are shown in Figures S1, S2, S3, S4, and S5. Proteins with significant (p≤0.05) probability-based MOWSE score are shown in Table S3. All together, we identified 27 novel HYPK-interacting proteins by pull-down coupled with mass spectrometry (MS) analysis using SCL from different cell lines as prey. In summary, we could identify 9 proteins using HeLa SCL, 12 proteins using Neuro2A SCL, 3 proteins using ST*Hdh^Q7^*/*Hdh^Q7^* SCL and 3 proteins using ST*Hdh^Q111^*/*Hdh^Q111^* SCL as prey. The detailed result is shown in Table 1.

**Table 1:** HYPK-interacting proteins identified in the present study and obtained earlier.

Gene identified (HUGO name)	HUGOsymbol	Gene ID (Human)	Source of protein	Validation method(s)
1. Huntingtin	HTT	3064	––	*Faber et. al, Hum Mol Genet. 1998, vol. 7, 1463–74*
2. Archain 1	ARCN1	372	SCL from HeLa (1D SDS-PAGE)	Not validated
3. Eukaryotic translationelongation factor 1 alpha 1	EEF1A1	1915	SCL from HeLa (1D SDS-PAGE)	Confocal microscopy and co-IP, identified in more than one experiment
4. Heat shock 70kDa protein 8	HSPA8	3312	SCL from HeLa and ST*Hdh^Q7^/Hdh^Q7^*(Both 1Dand 2D SDS-PAGE)	Confocal microscopy and co-IP, identified in more than one experiment
5. Lamin B2	LMNB2	84823	SCL from HeLa (1D SDS-PAGE)	Confocal microscopy and co-IP
6. Lamin B receptor	LBR	3930	SCL from HeLa (1D SDS-PAGE)	Not validated
7. Triosephosphate isomerase 1	TPI1	7167	SCL from HeLa (1D SDS-PAGE)	Not validated, identified in more than one experiment
8. Serologically defined coloncancer antigen 1	SDCCAG1	9147	SCL from HeLa (1D SDS-PAGE)	Not validated
9. Leukocyte receptor cluster(LRC) member 8	LENG8	114823	SCL from HeLa (1D SDS-PAGE)	Not validated
10. IKBKB interacting protein	IKBIP	121457	SCL from HeLa (1D SDS-PAGE)	Not validated
11. Calmodulin 1	CALM1	801	SCL from Neuro2A (1D SDS-PAGE)	Confocal microscopy and co-IP, identified in more than one experiment
12. Cadherin 11	CDH11	1009	SCL from Neuro2A (1D SDS-PAGE)	Not validated
13. Calreticulin	CALR	811	SCL from Neuro2A (1D SDS-PAGE)	Not validated
14. Centrosomal protein290 kDa	CEP290	80184	SCL from Neuro2A (1D SDS-PAGE)	Not validated, identified in more than one experiment
15. Zinc finger protein 462	ZNF462	58499	SCL from Neuro2A (1D SDS-PAGE)	Not validated
16. Zinc finger protein 100	ZNF100	163227	SCL from Neuro2A (1D SDS-PAGE)	Not validated
17. Zinc finger protein 516	ZNF516	9658	SCL from Neuro2A (1D SDS-PAGE)	Not validated
18. Kinesin family member 20B	KIF20B	9585	SCL from Neuro2A (1D SDS-PAGE)	Not validated
19. Heat shock protein 90kDaalpha (cytosolic), class Bmember 1	HSP90AB1	3326	SCL from ST*Hdh^Q7^*/*Hdh^Q7^*(1D SDS-PAGE)	Not validated, identified in more than one experiment
20. Myomesin family, member 3	MYOM3	127294	SCL from ST*Hdh^Q7^*/*Hdh^Q7^*(1D SDS-PAGE)	Not validated
21. Phosphoglycerate mutase 1(brain)	PGAM1	5223	SCL from ST*Hdh^Q111^*/*Hdh^Q111^* (1D SDS-PAGE)	Not validated
22. NEDD4 binding protein 1	N4BP1	9683	SCL from Neuro2A (2D SDS-PAGE)	Not validated
23. ATPase, H+ transporting,lysosomal V0 subunit a4	ATP6V0A4	50617	SCL from Neuro2A (2D SDS-PAGE)	Not validated
24. Glutamate dehydrogenase 1	GLUD1	2746	SCL from Neuro2A (2D SDS-PAGE)	Not validated
25. Glutamate dehydrogenase 2	GLUD2	2747	SCL from Neuro2A (2D SDS-PAGE)	Not validated
26. Serrate RNA effector moleculeHomolog (Arabidopsis)	SRRT	51593	SCL from ST*Hdh^Q111^/Hdh^Q111^* (2D SDS-PAGE)	Not validated
27. Protein phosphatase 6,regulatory subunit 2	PPP6R2	9701	SCL from ST*Hdh^Q111^/Hdh^Q111^* (2D SDS-PAGE)	Not validated
28. Non-metastatic cells 2,protein (NM23B)	NME2	4831	SCL from ST*Hdh^Q7^/Hdh^Q7^* (2D SDS-PAGE)	Not validated
29. Vimentin	VIM	7431	––	Confocal microscopy and co-IP
30. Heat shock transcriptionfactor 1	HSF1	3297	––	Confocal microscopy and co-IP
31. Heat shock 70kDaprotein 1A	HSPA1A	3303	––	Confocal microscopy and co-IP
32. Heat shock 27kDa protein 1	HSPB1	3315	––	Confocal microscopy and co-IP
33. Myeloid leukemia factor 1	MLF1	4291	––	Confocal microscopy and co-IP
34. Myeloid leukemia factor 2	MLF2	8079	––	Confocal microscopy and co-IP
35. DnaJ (Hsp40) homolog, subfamily B, member 3	DNAJB3	414061	––	Confocal microscopy and co-IP
36. v-relreticuloendotheliosisviral oncogenehomolog A (avian)	RELA	5970	––	Confocal microscopy and co-IP
37. Tumor protein p53	TP53	7157	––	Co-localization and co-IP
38. N(alpha)-acetyltransferase 10, NatA catalytic subunit	NAA10	8260	––	Anderson et al., Molecular and Cellular Biology (2010), Vol. 30, No. 8, p. 1898–1909
39. N(alpha)-acetyltransferase 15, NatA auxiliary subunit	NAA15	80155	––	Same as above
40. Heat shock 70kDa protein 14	HSPA14	51182	––	*Otto et al., PNAS, July 19, 2005, vol. 102, no. 29, p. 10064–10069*
41. DnaJ (Hsp40) homolog, subfamily C, member 2	DNAJC2	27000	––	Same as above
42. Chromodomain helicase DNA binding protein 3	CHD3	1107	––	*Stelzl et al., 2005, Cell, Vol.122, 957–968*
43. Group-specific component (vitamin D binding protein)	GC	2638	––	Same as above
44. MyoD family inhibitor	MDFI	4188	––	Same as above
45. Proteasome (prosome, macropain) activator subunit 3 (PA28 gamma; Ki)	PSME3	10197	––	Same as above
46. Quaking homolog, KH domain RNA binding (mouse)	QKI	9444	––	Same as above
47. RNA binding protein with multiple splicing	RBPMS	11030	––	Same as above
48. Rhoxhomeobox family, member 2	RHOXF2	84528	––	Same as above
49. TH1-like (Drosophila)	TH1L	51497	––	Same as above

HYPK-interacting proteins identified by us from pull-down of purified 6HN-HYPK protein as bait with various mammalian SCL as prey (no. 2–28), the HTT-interacting proteins as HYPK partners (no. 29–37) and previously reported HYPK-interacting partners (no. 1 and 38–49).

### Validation of Novel Interactions of HYPK Identified in MS Experiments

**Figure 2:**
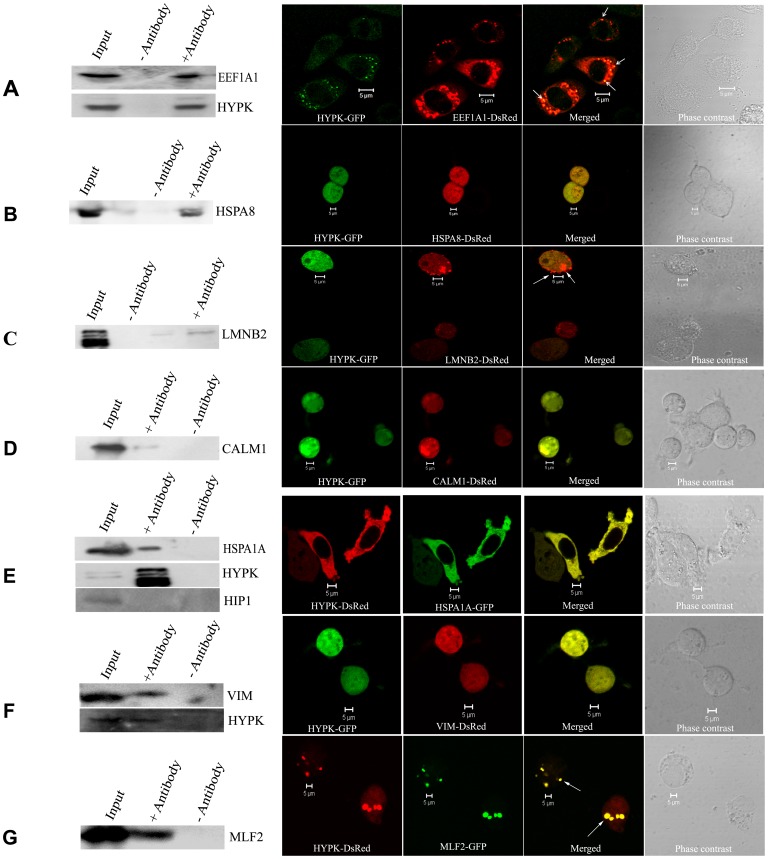
Validation of interaction by co-immunoprecipitation (co-IP) and confocal microscopy. Validation of interaction between HYPK and EEF1A1 by co-IP using anti-HYPK antibody (endogenous EEF1A1 was detected) along with subcellular co-localization of HYPK-GFP with EEF1A1-DsRed (***A***). The interaction of HYPK with HSPA8, LMNB2, CALM1, HSPA1A, VIM and MLF2 are shown (***B, C, D, E, F*** and ***G*** respectively). The co-IP of Neuro2A SCL with anti-HYPK antibody precipitated endogenous HSPA1A (immunoblot probed with anti-HSPA1A; ***E***). Anti-HYPK antibody was used to immunoprecipitate the HYPK-complex from Neuro2A SCL overexpressing HSPA8-DsRed (**B**), LMNB2-DsRed (***C***), CALM1-DsRed (***D***) or VIM-DsRed (***F***) and the immunoblots were probed with anti-DsRed antibody. Interaction between HYPK and MLF2 was confirmed by probing the HYPK-immunoprecipitated complex with anti-GFP antibody (**G**). The co-IP blots shown in ***A, E*** and ***F*** were re-probed with anti-HYPK antibody confirming HYPK in the immunoprecipitate. The dots showing co-localization of HYPK with EEF1A1 (***A***), LMNB2 (***C***) and MLF2 (***G***) are indicated by arrows. The immunoblot showing interaction between endogenous HYPK and endogenous HSPA1A was stripped and re-probed with anti-HIP1 antibody. The absence of HIP1 in the immunoprecipitate confirmed that HYPK did not interact with HIP1 (***E***). Scale bars (5 µm) for the confocal images are indicated in each panel.

We validated the interactions of HYPK with EEF1A1/EF1α, HSPA8/Hsc70, LMNB2/LaminB2 and CALM1/Calmodulin (Table 1, #3, 4, 5 and 11 respectively) by co-IP assay in Neuro2A cell lines. Co-localizations of HYPK with these proteins in Neuro2A cells were also detected using confocal imaging. Representative results for co-IP of endogenous HYPK with endogenous EEF1A1 along with co-localization of HYPK-GFP with EEF1A1-DsRed are shown in Figure 2, panel A, where co-localized dots are visible in HYPK-EEF1A1 merged picture (R^2^ = 0.88). The co-IP blot was also re-probed with anti-HYPK antibody to confirm the presence of HYPK in the immunoprecipitated complex. Similar results obtained with three other proteins, *viz.,* HSPA8, LMNB2 and CALM1 are shown in Figure 2, panels B, C and D respectively. In all 3 cases, blots for HYPK-immunoprecipitates were probed with anti-DsRed antibody. In case of confocal imaging of LMNB2-DsRed with HYPK-GFP, co-localization was observed only in the dotted structures of LMNB2-DsRed. The squared Pearson correlation coefficient values (R^2^) for all co-localization experiments are shown in Table 2. Although co-localization between two cytosolic proteins may not be used as a method to validate their interaction, these positive imaging results supported our results of co-IP.

**Table 2:** Squared values of the Pearson Correlation coefficient (R_p_) for determination of co-localization of HYPK with its interacting partners.

Protein Name	R^2^ values
EEF1A1	0.88
HSPA8	0.96
LMNB2	0.77
CALM1	0.93
HSPA1A	0.96
HSPB1	0.85
HSF1	0.60
VIM	0.65
TP53	0.86
RELA	0.53
DNAJB3	0.97
MLF1	0.65
MLF2	0.84
HIP1	0.37

Extent of co-localization of HYPK with 14 interacting proteins as obtained from confocal imaging studies. The R^2^ values were analyzed to validate whether the co-localization with HYPK was significant. Values greater than or equal to 0.5 were considered to be significant for these co-localization studies.

### Identification of HTT-interacting Proteins as Interacting Partners of HYPK

Among the HYPK-interacting proteins identified (as mentioned above), HSPA8 [22], EEF1A1 [23] and CALM1 [24] have been previously reported to interact with HTT and were also reviewed recently [25]. Based on these observations, we hypothesized that other HTT-interacting proteins, especially the chaperones, might interact with HYPK. To test the hypothesis, we performed co-IP and also checked co-localization of HYPK with known HTT-interacting chaperones and non-chaperone proteins like HSP70/HSPA1A, HSP27/HSPB1, HCG3/DNAJB3, MLF1, MLF2, HIP1, TP53, p65/RELA subunit of NFκB [25] and the HD-associated protein VIM [26]. It is known that HSF1 regulates the expression of heat shock proteins and also interacts with other chaperones. Exogenous expression of this protein reduces the aggregates formed by mutant HTT [27], [28]. Thus, we also tested whether HYPK interacts with HSF1. We found that except for HIP1, a HTT-interacting protein, all the other HTT-interacting partners interacted with HYPK. Among these, interaction of endogenous HYPK with endogenous HSPA1A, VIM-DsRed and MLF2-GFP were confirmed by co-IP (Figure 2, panel E, F and G respectively). For all these three cases, anti-HYPK antibody was used to immunoprecipitate the endogenous HYPK-complex, and the blots were probed with anti-HSPA1A, anti-DsRed and anti-GFP antibodies respectively. The blots were stripped and re-probed with anti-HYPK antibody; results showed the presence of HYPK in the immunoprecipitated complex. Significant co-localizations of HYPK with these three proteins were also observed in confocal imaging studies. The blot for HSPA1A was further probed with anti-HIP1 antibody which showed lack of interaction between HYPK and HIP1 (Figure 2, panel E). Results obtained with the other HTT-interacting proteins mentioned above are shown in Figure S6 and all R^2^ values for co-localization are shown in Table 2. Thus we further identified 9 additional HYPK-interacting proteins (Table 1, #29–37). To exclude the possibility that HYPK non-specifically interacts with other proteins, interaction of HIP1 with HYPK was further checked by independent IP experiments, pulling-down the HYPK-protein complex and probing with anti-HIP1 antibody. However, we could not find any interaction between HYPK and HIP1. HYPK and HIP1 were also not found to be co-localized (R^2^ = 0.37) (Figure S6, panel G). This result suggests strongly that HYPK interacts specifically with its partners reported here.

### Complex Formation by HYPK with its Interacting Partners

**Figure 3:**
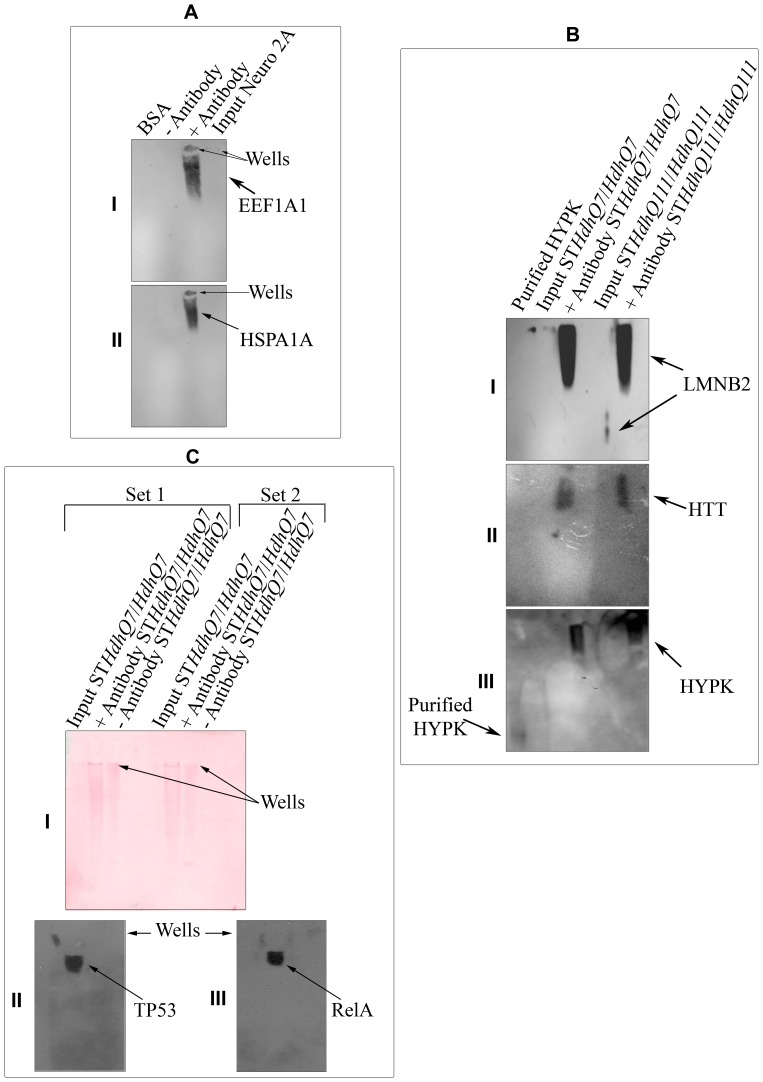
Formation of high molecular weight HYPK-complex. High molecular weight HYPK-complex formation by endogenous HYPK with EEF1A1 (***AI***) and HSPA1A/HSP70 (***AII***) in Neuro2A cells using native-PAGE western analysis; results obtained by similar experiments in ST*Hdh^Q7^*/*Hdh^Q7^* and ST*Hdh^Q111^*/*Hdh^Q111^* with LMNB2 (***BI***) and HTT (***BII***); result obtained by re-probing the blot shown in ***B*** with anti-HYPK antibody is shown in ***BIII***, showing the difference in migration of purified HYPK and complex-bound HYPK; similar co-IP experiments in ST*Hdh^Q7^*/*Hdh^Q7^* (***C***) along with Ponceau staining of the nitrocellulose membrane (***CI***). The blot is probed with anti-TP53 (***CII***) and anti-RELA (***CIII***) antibodies. In all the cases, anti-HYPK antibody was used to pull-down and the immunoprecipitate elute was analyzed to detect the interacting proteins. The loading wells in ***A*** and ***C*** are marked by arrows.

We performed IP of endogenous HYPK followed by native PAGE-western blot analysis of the immunoprecipitated complex as described earlier to detect protein complexes formed by HYPK. When Neuro2A cell extract was immunoprecipitated by anti-HYPK antibody, we detected EEF1A1 and HSPA1A as members of the high molecular weight complex at the top of the gel (Figure 3, panels AI and AII respectively). When ST*Hdh^Q7^*/*Hdh^Q7^*and ST*Hdh^Q111^*/*Hdh^Q111^* cell extracts were immunoprecipitated in a similar way, we detected HTT (Figure 3, panel BII) and LMNB2 (Figure 3, panel BI) in the high molecular weight complex. Endogenous LMNB2 was identified at a low molecular weight position. When re-probed with anti-HYPK antibody, we found HYPK in the same complex with HTT and LMNB2, whereas purified recombinant HYPK was observed at a much low molecular weight position (Figure 3, panel BIII, first lane from left). The endogenous TP53 and RELA were also found to be components of the HYPK-complex (Figure 3, panels CII and CIII) when cell extract of ST*Hdh^Q7^*/*Hdh^Q7^* was immunoprecipitated. Ponceau S staining of the membrane after western transfer shows that the proteins were not stuck to the wells but rather were present throughout the lanes (Figure 3, panel CI). These results showed that in different neuronal cells, HYPK could form complex with interacting partners (HTT and LMNB2), (EEF1A1 and HSPA1A), and (TP53 and RELA).

### Enrichment of GO Terms with the HYPK-interacting Proteins

Likelihood that a protein participates in a specific function, biological process or pathway increases if the interacting partner(s) of that particular protein is known to be involved in the same process or pathway [29]. Thus to get more information about function(s) of HYPK, we analyzed 49 HYPK-interacting proteins (37 of these identified in this study and the other 12 identified earlier by different investigators) for the enrichment of GO terms using the freely available software, GeneCodis2 (http://genecodis.dacya.ucm.es/analysis/) [18], [19] as described in the ***Materials and Methods*** section. Out of 49 HYPK-interacting proteins, 45 were over-represented in 47 different GO molecular function classes (corrected hyper geometric significance of ≤0.05). The GO term protein binding (MF, GO: 0005515) was found to be enriched with 29 out of the 47 HYPK-interacting protein-coding genes compared to 6618 among 29,095 genes coded by human genome, as cataloged in GeneCodis2. Similarly, the GO term unfolded protein binding (GO: 0051082) was significantly enriched with HYPK-interacting proteins DNAJC2, HSP90AB1, HSPA8 and CALR while DNA binding (GO:0003677) was found to be enriched with ZNF462, CHD3, NFκB (p65)/RELA, DNAJC2, MLF1, LBR, ZNF516, ZNF100, CALR and TP53. The complete list of these proteins is shown in Table S4A.

Several GO terms (135) describing the biological processes were also found to be significantly enriched with the interacting partners of HYPK (Table S4B). The GO biological process DNA-dependent transcription (GO: 0006355) were found to be enriched with TP53, CHD3, HSF1, RHOXF2, ZNF100 and CALR and cell cycle arrest (GO: 0007050) were enriched with TP53, MLF1, KIF20B and CALR. Such enrichment of various GO terms with the HYPK-interacting proteins indicates that HYPK through its interaction with other proteins might participate in various biological processes including DNA binding, protein folding, response to unfolded protein, apoptosis, cell cycle arrest and transcription. It is important to validate such prediction by further experiments.

### Pathway Analysis of HYPK-interacting Proteins

Biological pathway is defined as the series of sequential causal interactions that mediate the signals, generally from outside the cells, to travel within, and sometimes communicate between the cells and essentially control cellular behaviors. If a protein is involved in a specific biological pathway, its interacting partner is likely to participate in the same.

KEGG pathway analysis of HYPK-interacting proteins using GeneCodis2 revealed that 16 different pathways were significantly enriched with 45 proteins except PPP6R2, DNAJB3, NAA10, NAA15 and C15orf63/HYPK. For example, MAPK signaling pathway (KEGG, 04010) was significantly enriched with HYPK-interacting proteins RELA, HSPB1, TP53 and HSPA8 (p = 0.007). Other pathways significantly enriched with the HYPK-interacting proteins included Glycolysis/Gluconeogenesis (KEGG, 00010), apoptosis (KEGG, 04210) and pathways in cancer (KEGG, 05200). The result is shown in Table S4C. The Reactome pathway analysis revealed that HYPK-interacting partners were associated with pathways like regulation of mRNA stability, metabolism of RNA, carbohydrate metabolism, ubiquitination, muscle contraction, DNA damage response, TP53 stabilization, etc. The enrichment of 16 specific pathways in the Reactome database with 49 HYPK-interacting partners is provided in Table S4D. Thus, bioinformatics analyses indicate that HYPK interactome participates in multiple molecular functions, biological processes and pathways.

### Subcellular Localization and Expressions of Genes Coding for HYPK-interacting Partners in Brain

For functional interaction to be possible, HYPK and its interacting partner(s) should be expressed not only in the same cell/tissue but also in the same cellular compartment. HYPK is a HTT-interacting chaperone protein. So, for functional relevance in HD biology, both HYPK and its interacting partners should be expressed in the human brain. To check this out, we utilized various databases like BioMart, TiGER and Nextprot that catalogue the expression of genes. Among 50 proteins (49 HYPK-interacting proteins and HYPK itself), expressions of 48 in the brain were described in at least one of the three databases (Table S5). Since expression of HYPK was identified only in TiGER, we searched other resources and observed that the expression of HYPK/C15orf63 (Affimatrix ID 218680_x_at) has been identified in human brain in many studies and also reported in Gene Expression Omnibus (GEO) database. Similarly, in GEO database, expressions of GC and ATP6V0A4 were reported. Thus, HYPK and its interacting partners are likely to be expressed in human brain, although to different extents. Localizations of HYPK and its interacting partners were also determined using the Nextprot database. HYPK was found to be a cytosolic protein according to this database. It has been reported earlier that HYPK is associated with ribosomes [4], [5]. Among others, information on LENG8, DNAJB3 and MYOM3 were not available in the database. Sixteen proteins, including HYPK, were observed only in cytoplasm including mitochondria, golgi and endoplasmic reticulum. Group-specific component (vitamin D binding protein) GC is a secreted protein. Eleven proteins were identified only in nucleus and 19 were detected both in cytoplasm and nucleus (Table S5). Thus, 34 proteins all together could be identified in cytoplasm. Although requires further evidence, it is possible that HYPK might move to the nucleus by interacting with the proteins that can shuttle between nucleus and cytoplasm. It remains to be found out whether HYPK can also be localized within mitochondria. The expression of HYPK and its interacting proteins in the brain, and localization of many proteins in cytoplasm indicate that HYPK interactome identified in this article could be functional in brain and that most of such interactions possibly occur in cytoplasm.

### Downregulation of HYPK in Neuronal Cell Lines

**Figure 4:**
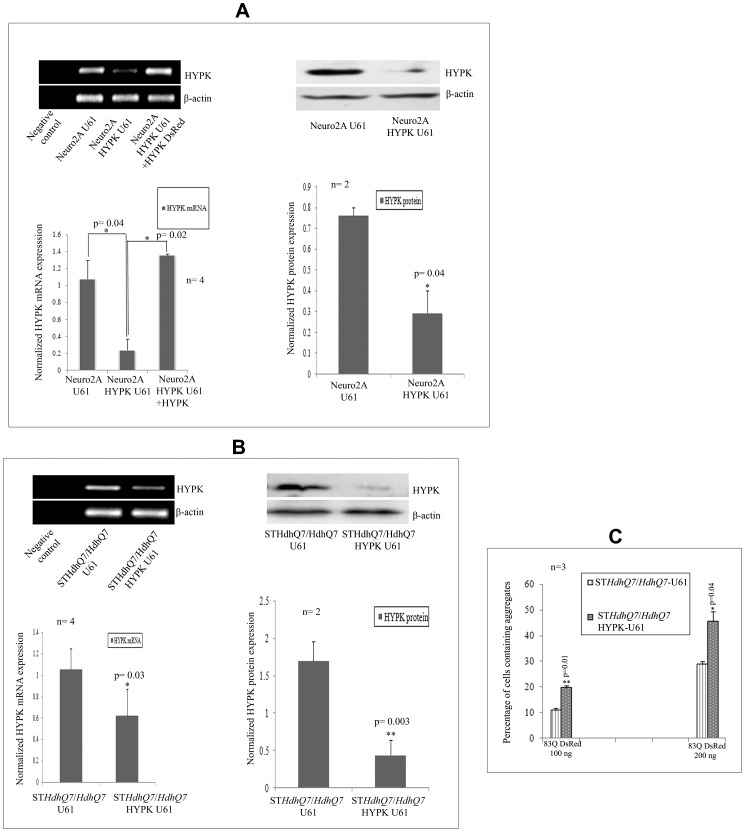
Preparation of HYPK knock-down neuronal cell lines using the antisense construct (HYPK-U61). Validation of HYPK knock-down in mRNA and protein levels in Neuro2A (***A***) and ST*Hdh^Q7^*/*Hdh^Q7^* (***B***) cell lines. Expression of both mRNA and protein levels were measured by RT-PCR or SDS-PAGE western analysis in these HYPK knocked-down stable mouse cell lines. Upon exogenous expression of 83Q-DsRed in a dose dependent manner (100 ng and 200 ng), the percentage of cells containing mutant HTT aggregates in presence and absence of endogenous HYPK in ST*Hdh^Q7^*/*Hdh^Q7^* cell lines are compared (***C***). The ‘n’ and ‘p’ values for Student’s two-tailed t test are indicated in the bar diagrams along with the mean and standard deviation.

To test experimentally whether HYPK participates in cell growth, cell cycle regulation, misfolded protein responses and cell death, we knocked down or overexpressed HYPK in cells and studied the responses. We used antisense *HYPK* as described in the ***Materials and Methods*** section to downregulate HYPK in ST*Hdh^Q7^*/*Hdh^Q7^*and Neuro2A cells. In Neuro2A cells, expression of the antisense *HYPK* construct significantly decreased HYPK expression at both mRNA (p = 0.04, n = 4) and protein (p = 0.04, n = 2) levels (Figure 4, panel A). Significant downregulation of HYPK both in the mRNA (p = 0.03, n = 4) and protein (p = 0.003, n = 2) levels was also observed in ST*Hdh^Q7^*/*Hdh^Q7^* cells (Figure 4, panel B).To find out the specificity of such downregulation, we overexpressed HYPK-GFP in these HYPK downregulated Neuro2A cells and detected expression of HYPK mRNA (Figure 4A, lane 3 from the left). Exogenous HYPK could significantly recover the decreased expression of HYPK at mRNA level (p = 0.02, n = 4).

We have shown earlier that overexpression of HYPK in neuronal as well as non-neuronal cells reduces the aggregates formed by the N-terminal mutant HTT coded by exon1 of *HTT* gene with expanded CAG repeats [2]. It is thus expected that reduction of endogenous HYPK would increase aggregates formed by the N-terminal mutant HTT. Expression of N-terminal HTT with 83Q in ST*Hdh^Q7^*/*Hdh^Q7^* cells increased aggregate formation in a dose dependent manner (Figure 4, panel C). When we transfected ST*Hdh^Q7^*/*Hdh^Q7^*-U61 (control) and ST*Hdh^Q7^*/*Hdh^Q7^* HYPK-U61 cells with 100 ng of 83Q-DsRed, we observed a significant 9% increase (p = 0.01, n = 3) in the number of aggregates in cells with reduced HYPK expression. Number of aggregates was increased by 16.6% (p = 0.04, n = 3) upon transfection with 200 ng of 83Q-DsRed. Similar result was also reported earlier in human non-neuronal cells, where reduction of endogenous HYPK increased the aggregates formed by mutant HTT [4]. This result further confirmed that antisense construct reduced the endogenous expression of HYPK.

### HYPK Downregulation Reduces Cell Growth

**Figure 5:**
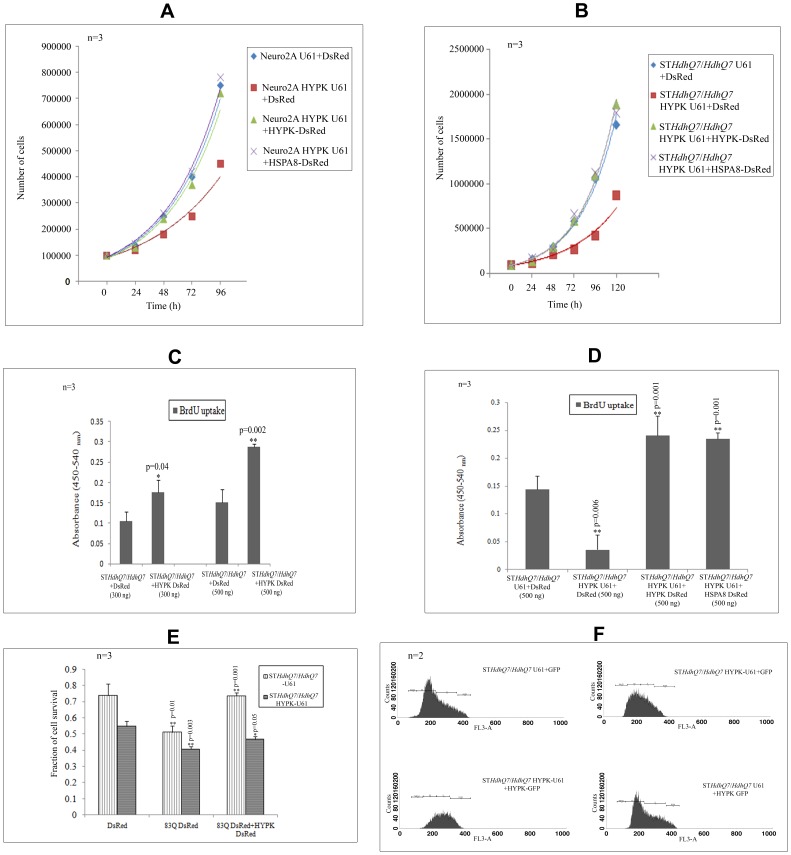
Cell growth and cell survival in presence and absence of HYPK. Comparison of the growth curves of control and HYPK downregulated Neuro2A (***A***) and ST*Hdh^Q7^*/*Hdh^Q7^* (***B***) cell lines and effect of exogenous expression of HYPK-DsRed and HSPA8-DsRed in these HYPK knocked-down neuronal cell lines. Comparison of BrdU incorporation in ST*Hdh^Q7^*/*Hdh^Q7^* cells overexpressing DsRed and HYPK-DsRed in a dose dependent manner (transfection with 300 ng and 500 ng of plasmid) (**C**). The difference in BrdU incorporation in ST*Hdh^Q7^*/*Hdh^Q7^* cells in absence of HYPK and in presence of HYPK as well as HSPA8 (500 ng) are shown (***D***). The effect of mutant Htt (83Q-DsRed) on cell survival of control and HYPK downregulated ST*Hdh^Q7^*/*Hdh^Q7^* cells and its effect in presence of exogenous addition of HYPK-DsRed in these cells are shown (***E***). Flow cytometry analysis showing distribution of ST*Hdh^Q7^*/*Hdh^Q7^* cells in different phases of cell cycle (upon 7-AAD staining) in presence and absence of HYPK (**F**). The cell cycle analysis was performed using CellQuest Pro software as described in ***Materials and Methods*** section.The ‘n’ and ‘p’ values for Student’s two-tailed t test are indicated in the bar diagrams along with the mean and standard deviation. The distributions of cell population in different phases are provided in Table 3.

Neuro2A cells and ST*Hdh^Q7^*/*Hdh^Q7^* cells with reduced HYPK expressions grew slowly compared to that of the control Neuro2A-U61 and ST*Hdh^Q7^*/*Hdh^Q7^*-U61 cells respectively. The average time required for doubling the cell numbers in Neuro2A-U61 and ST*Hdh^Q7^*/*Hdh^Q7^*-U61 cells was ∼28 h and ∼30 h respectively, while in cells with reduced HYPK, these values were ∼34 h and ∼38 h respectively. Thus, reduction of endogenous HYPK slowed down cell growth. Exogenous expression of HYPK-GFP recovered the reduced cell growth observed in HYPK downregulated cells. The doubling time for Neuro2A and ST*Hdh^Q7^*/*Hdh^Q7^* cells co-expressing HYPK-U61 and HYPK-GFP was found to be ∼27 h and ∼31 h respectively. HSPA8, a HYPK-interacting chaperone, could also recover the defects in cell growth caused by HYPK knock-down. Doubling time for HYPK downregulated Neuro2A and ST*Hdh^Q7^*/*Hdh^Q7^* cells overexpressing HSPA8-DsRed was ∼27 h and ∼30 h respectively (Figure 5, panels A and B). The mean values and standard deviations for each data point are provided in Tables S6A and S6B.

**Table 3:** Distribution of cell populations in different cell cycle phases in presence and absence of HYPK in ST*Hdh^Q7^* ^/^ *Hdh^Q7^* cell lines.

	G0/G1	S	G2/M	Sub G1
ST*Hdh^Q7^*/*Hdh^Q7^* U61+GFP	55.025±0.3	35.645±0.2	6.01±0.1	0.495±0.5
ST*Hdh^Q7^*/*Hdh^Q7^* HYPK-U61+GFP	54.88±5.2	10.495±0.2	1.85±1.8	31.485±4.7
ST*Hdh^Q7^*/*Hdh^Q7^* HYPK-U61+HYPK-GFP	49.88±0.5	29.63±6.8	15.2±9.8	3.015±3.8
ST*Hdh^Q7^*/*Hdh^Q7^* U61+HYPK-GFP	52.34±0.7	36.65±2.1	8.59±2.0	0.18±0.2

Percentage of cell population in different phases of cell cycle (as analyzed by CellQuest Pro software) in presence and absence of HYPK as observed with ST*Hdh^Q7^*/*Hdh^Q7^*cell lines.

BrdU cell proliferation assay revealed that incorporation of BrdU increased significantly upon transfection with 300 ng of HYPK-DsRed in the parental ST*Hdh^Q7^*/*Hdh^Q7^*cells as compared to cells transfected with the same amount of DsRed vector (p = 0.04, n = 3). Such significant increase in BrdU incorporation was also observed when we overexpressed 500 ng of HYPK-DsRed in ST*Hdh^Q7^*/*Hdh^Q7^*cells (p = 0.002, n = 3) (Figure 5, panel C). We also observed that downregulation of HYPK in ST*Hdh^Q7^*/*Hdh^Q7^* cells reduced cell proliferation significantly (p = 0.006, n = 3). This reduction in cell proliferation could also be rescued by exogenous expression of HYPK-DsRed (p = 0.001, n = 3) or HSPA8-DsRed (p = 0.001, n = 3) (Figure 5, panel D). Thus the difference in BrdU incorporation in control and HYPK downregulated ST*Hdh^Q7^*/*Hdh^Q7^* cells supported our observations shown in panels A and B of Figure 5.

### Role of HYPK in Cell Death

We further tested whether reduced expression of HYPK affected cell death induced by N-terminal mutant HTT with 83 Q repeats. We have shown earlier that overexpression of HYPK protected cells from death induced by mutant N-terminal HTT [2]. Reduction of endogenous HYPK increased the toxicity (decreased the survival) in ST*Hdh^Q7^*/*Hdh^Q7^* cells. In MTT assay, cell survival was about 73% in ST*Hdh^Q7^*/*Hdh^Q7^* cells expressing the control vector (U61), while this value was about 55% in ST*Hdh^Q7^*/*Hdh^Q7^* cells expressing HYPK-U61 (antisense construct). This difference was statistically significant (p = 0.02, n = 3). Overexpression of 83Q-DsRed in ST*Hdh^Q7^*/*Hdh^Q7^* cells with endogenous HYPK reduced cell survival to 51% (p = 0.01, n = 3) and in ST*Hdh^Q7^*/*Hdh^Q7^* cells with decreased HYPK to 41% (p = 0.003, n = 3). Co-expression of the N-terminal mutant HTT and HYPK-DsRed in ST*Hdh^Q7^*/*Hdh^Q7^* cells recovered the toxicity significantly (73% cell survival; p = 0.001, n = 3). Overexpression of HYPK-DsRed in HYPK downregulated ST*Hdh^Q7^*/*Hdh^Q7^* cells increased survival from 41% to 47% (p = 0.05, n = 3). This result, shown in Figure 5, panel E, indicated that HYPK is involved in the recovery of cell death under normal condition or general stress induced by the mutant HTT.

### Role of HYPK in Cell Cycle Regulation

The GO terms related to cell cycle were found to be significantly enriched with HYPK-interacting proteins (Table S4B). To test the role of HYPK in cell cycle regulation, we analyzed the distribution of cells in different phases of cell cycle. In asynchronously growing ST*Hdh^Q7^*/*Hdh^Q7^* cells transfected with the control vectors, proportion of cells in G0/G1, S, G2/M and sub G1 were (55.0±0.3%), (35.6±0.2%), (6.0±0.1%) and (0.5±0.5%) respectively. In cells with reduced HYPK (ST*Hdh^Q7^*/*Hdh^Q7^*-HYPK-U61), the proportion of cells in sub G1 increased significantly (p = 0.01, n = 2), while that in S phase decreased significantly (p<0.0001, n = 2). This increase in the sub G1 cells indicates that these cells were undergoing apoptosis. These abnormalities could be partially rescued by overexpressing HYPK-GFP in these HYPK knocked-down cells (Table 3). Results of a typical experiment is shown in Figure 5, panel F. It is interesting to note that reduction of endogenous HYPK resulted in a decreased number of cells in S phase. This result is similar to that observed in BrdU incorporation assay where exogenous expression of HYPK was able to rescue the observed cell cycle abnormalities in cells with reduced HYPK. This result showed that HYPK was able to regulate distribution of cells in different phases of cell cycle and supported the inferences made by enrichment analysis of the HYPK-interacting proteins in different biological processes described above. Similar observation of accumulation of cells in sub G0/G1 phase by downregulating HYPK has also been made earlier in HeLa cells [4]. Additionally, we showed in this article that distribution of cells in different phases of cell cycle was also altered in presence and absence of HYPK.

### Recovery of Heat-stressed Luciferase Activities in Presence and Absence of HYPK

The GO analysis of HYPK-interacting partners revealed that GO terms for molecular functions like protein folding (GO: 0006457) and response to unfolded protein (GO: 0006986) were enriched significantly (Table S4A). Thus, we tested whether HYPK is involved in unfolded protein response using the luciferase reporter assay after heat shock following the methods described earlier [2]. We also tested whether some of the interacting partners of HYPK were involved in such processes.

**Figure 6:**
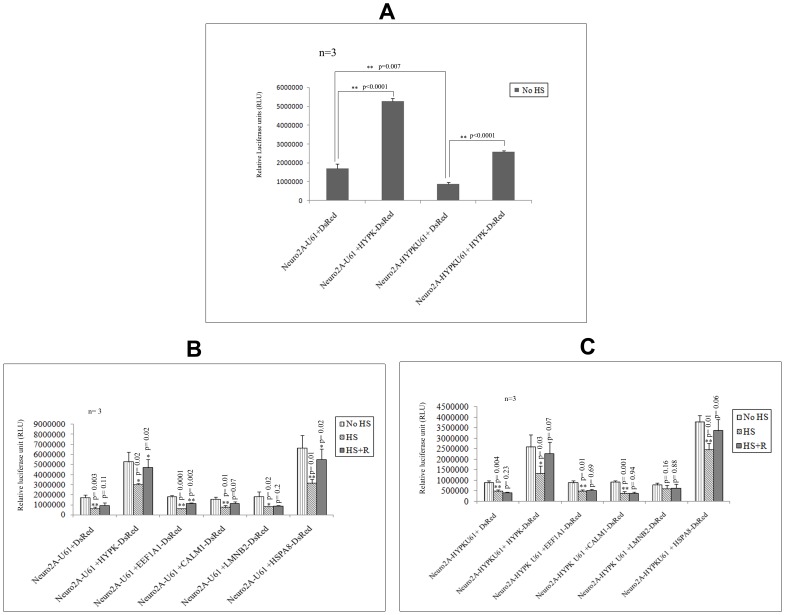
Effect of HYPK on recovery of heat denatured luciferase activity. The *in vivo* chaperone activities of Neuro2A-U61 and Neuro2A HYPK-U61 cells in presence and absence of HYPK (before administration of heat shock) are compared (***A***). As mentioned in the ***Materials and Methods*** section, the cells were subjected to heat shock at 43°C for 1 h (HS) and re-incubated at 37°C CO_2_ incubator for the next 6h (HS+R). In every case, heat shock was administered 24h post-transfection of Tet^+^Luc+ve cells with experimental constructs. Comparison of luciferase activities in Neuro2A-U61 cells in presence and absence of HYPK-DsRed in No HS, immediately after heat shock (HS) and HS+R conditions (**B**). These luciferase activities were also compared upon overexpression of HSPA8-DsRed and 3 other HYPK partners in Neuro2A-U61 cells (***B***). Such drop and recovery of the luciferase activities upon heat treatment of HYPK downregulated Neuro2A (Neuro2A-HYPK U61) cells and effect of HYPK and its interacting partners are shown (***C***). The ‘n’ and ‘p’ values for Student’s two-tailed t test are indicated in the bar diagrams along with the mean and standard deviation.

As mentioned in the ***Materials and Methods*** section, Tet^+^ Luc+ve Neuro2A-U61 (control) and Tet^+^ Luc+ve Neuro2A-HYPK U61 (HYPK downregulated) cells were transfected with empty DsRed, HYPK-DsRed or other HYPK-interactors cloned in DsRed. We compared the luciferase activities of these cells in conditions (i) without heat shock (No HS), (ii) immediately after heat shock (HS) and (iii) 6 h of growth following heat shock (HS+R). Even without heat shock, the reporter luciferase activity was decreased significantly (p = 0.007, n = 3) in Neuro2A HYPK-U61 cells as compared to Neuro2A-U61 cells (Figure 6, panel A). Overexpression of HYPK-DsRed in Neuro2A-U61 cells resulted in a significant increase in the luciferase activity (p<0.0001, n = 3) as compared to that of DsRed transfected control (Neuro2A-U61) cells (Figure 6, panel A). When HYPK-DsRed was overexpressed in Tet^+^Luc+ve Neuro2A-HYPK U61 cells, the luciferase activity was also increased significantly (p<0.0001, n = 3) (Figure 6, panel A), compared to that obtained in HYPK downregulated cells.

Immediately after heat treatment (43°C for 1h), luciferase activities were decreased. We measured the decrease and recovery of luciferase activities in all the above mentioned conditions. As evident from Figure 6, panel B, immediately after heat shock (HS), the luciferase activity dropped to 35.4% (p = 0.003, n = 3) and 57.1% (p = 0.02, n = 3) of the initial values obtained without heat shock (No HS) in Neuro2A-U61 cells overexpressing DsRed and HYPK-DsRed respectively. After 6 h of recovery following heat treatment (HS+R), Neuro2A-U61 cells overexpressing DsRed and HYPK-DsRed recovered these drop in luciferase activities to about 19.7% (p = 0.11, n = 3) and 32.4% (p = 0.02, n = 3) of the values obtained immediately after heat shock (HS); suggesting the importance of HYPK in facilitating the refolding of heat-denatured luciferase (Figure 6, panel B). This result was further supported by the observation that in HYPK downregulated Neuro2A cell extract (Neuro2A-HYPK U61), the drop in the reporter luciferase activity immediately after heat shock (HS) (p = 0.004, n = 3) could not be recovered even after re-incubation for 6 h. However, when HYPK-DsRed was overexpressed in Neuro2A-HYPK U61 cells, we observed a marginal significant recovery (p = 0.07, n = 3) of 36% of the luciferase activity (Figure 6, panel C). HSPA8 was found to recover the drop in luciferase activities in both Neuro2A-U61 (35.7% recovery, p = 0.02, n = 3, Figure 6, panel B) and Neuro2A-HYPK U61 cell extracts (24.1% recovery, p = 0.06, n = 3, Figure 6, panel C). Two other interacting partners of HYPK, *viz.*, EEF1A1 and CALM1, when overexpressed in Neuro2A-U61 cells, were able to recover significantly the unfolded luciferase activities, although to different extents (Figure 6, panel B). However, the presence of HYPK was necessary for such recovery (Figure 6, panel C). The error bars in all the diagrams denote standard deviations, and Student’s unpaired two tailed t tests were performed to determine statistical significance as mentioned above.

## Discussion

In the present communication, we showed that HYPK, an intrinsically unstructured protein with chaperone-like activity, interacted with 36 novel proteins (Table 1, #2–37). HTT and 4 other proteins are known to interact with HYPK (Table 1, # 1 and 38–41), while 8 proteins were identified in yeast two-hybrid assays earlier by other investigators (Table 1, # 42–49). Among the 36 proteins identified as HYPK partners in this study, 13 were confirmed by other method(s) (3 proteins among these were identified in more than one independent experiment). Among the rest of the HYPK-interacting proteins, TPI1, CEP290 and HSP90AB1were also identified in more than one independent pull-down experiment (Table 1). Cell extracts from various mammalian cell lines *viz.,* HeLa, Neuro2A and ST*Hdh^Q7^*/*Hdh^Q7^* and ST*Hdh^Q111^*/*Hdh^Q111^* were used in pull-down and co-IP experiments. HYPK being a HTT-interacting protein, the interacting partners of HYPK identified using SCL from ST*Hdh^Q7^*/*Hdh^Q7^* and ST*Hdh^Q111^*/*Hdh^Q111^* as prey might have relevance to HD biology. It is interesting to mention here that HTT was also identified in a pull-down assay, however the MOWSE score (35) was not significant (p>0.05; data not shown). Molecular weight of full length HTT is ∼350 KDa. Resolution of such a large protein on SDS-PAGE may be difficult and the trypsin digestion-generated peptides are expected to yield less sequence coverage, resulting in a less than significant score.

Proteins, identified in a particular experiment by MS after pulling down the HYPK-bound partners from the column and separation on 1D or 2D polyacrylamide gels, are likely to form complex. However, these complexes may not originate from the same cellular compartment. We identified in different experiments that (HSPA1A and EEF1A1), (HTT and LMNB2) and (TP53 and RELA) could form complex with HYPK (Figure 3). HYPK and 35 other proteins are localized in the cytoplasm (Table S5). Thus, HYPK may form complexes in the cytoplasm. HYPK did not have any known nuclear localization signal. It remains unknown whether HYPK can form complexes with proteins in the nucleus. However, it is possible that some of the interacting proteins, which are localized in the cytoplasm and also in the nucleus, may transport HYPK to the nucleus. It is necessary to test this contention in future.

We observed that HYPK interacted with 4 chaperones, *viz.,* HSP90AB1 (Hsp90 family chaperone, PC00028), HSPA8, HSPA14 (Hsp70 family chaperone, PC00027) and DNAJC2 (subfamily not assigned). It is known that chaperones interact with each other. For example, HSP70 and chaperonin TriC/CCT are involved in folding newly synthesized proteins [30], while HSP70 and HSP90 are reported to be involved in refolding of denatured proteins [31]. Functional relevance of interactions of HYPK with other chaperones is not yet known.

Reduction of endogenous HYPK resulted in the impairment of refolding of heat-denatured reporter luciferase protein. Exogenous expressions of either HYPK or HSPA8 in the same cells could recover such defects. We have shown similar result earlier where overexpression of HYPK increases refolding of denatured proteins [2]. EEF1A1 and CALM1, interacting partners of HYPK, could enhance the ability of endogenous HYPK to refold the heat-denatured luciferase protein (Figure 6, panel B). Thus the interacting partners of HYPK, especially EEF1A1 and CALM1, are likely to be involved in response to unfolded proteins. This was also inferred from the enrichment analysis for GO terms.

Growth of neuronal cells, determined by counting number of cells at different time points, was reduced upon HYPK knock-down (Figure 5). It is also shown that incorporation of BrdU was reduced upon HYPK knock-down suggesting that DNA replication was impaired. This observation was also supported by the results that population of cells in S phase was decreased in cells with reduced HYPK. Such defects could be rescued either by HYPK or HSPA8. These effects could be mediated through increased protein synthesis or stabilizing the proteins involved in cell growth/cell cycle. Cells where the expression of HYPK was compromised were found to be blocked at sub G1 phase of cell cycle, indicating increased apoptosis [32]. A similar observation in HeLa cells has been published earlier [4]. Again this can be rescued by overexpression of HYPK. Similar effects of other chaperones on cell cycle, cell growth and apoptosis are known and reviewed [33], [34], [35], [36]. Interestingly, a recent study shows wild-type HTT to be localized in the mitotic spindle during mitosis and a decrease in wild-type HTT alters mitosis [37]. It will be worthwhile to test whether HYPK also localizes in such complexes and regulates mitosis. Further, kinesin family member 20B (KIF20B/M-phase phosphoprotein 1), identified as an interacting partner of HYPK (Table 1), mainly localizes to nuclei in interphase, but during mitosis, diffuses throughout the cytoplasm. In anaphase, it localizes to the midbody. It is proposed that KIF20B is involved in cytokinesis [38]. Thus HYPK may also contribute to cell growth by interacting with KIF20B. Reduction of HYPK resulted in increased apoptosis [4] and toxicity (shown in the present study). It can be speculated that HYPK could be an anti-apoptotic/pro-survival protein. It has been shown that HTT itself is an anti-apoptotic protein as *htt* knocked-out mice is embryonic lethal with increased markers of apoptosis in brain [39], [40]. The significance of interaction of HYPK with transcription regulators like HSF1, TP53 and RELA requires further investigations.

Transcription deregulation, enhanced apoptosis, defects in proteasomal degradation, abnormalities in Glycolysis/Gluconeogenesis are observed in HD [33], [34]. Some of these pathways known to alter in HD were enriched with several HYPK-interacting proteins (Table S4C and Table S4D). This result indicates that HYPK together with its interacting partners might be involved in HD pathogenesis. HYPK was considered to be a hypothetical protein that interacts with HTT in yeast two-hybrid assay and was not confirmed further until 2008. In last few years, several functions of HYPK such as chaperone-like activity, apoptosis, protein synthesis, cell cycle regulation and protein modification have been observed [3], [4], [5], [13]. Identification of 36 new interacting partners of HYPK, their involvement in relevant biological processes and pathways related to HD pathogenesis and experimental verification of some of the processes indicate that HYPK in collaboration with its interacting partners might be involved in biological processes and pathways crucial for HD pathology.

## Supporting Information

Figure S1 **MALDI-MS identification of HSPA8 as HYPK-interacting partner.**
(PDF)

Figure S2 **MALDI-MS identification of LMNB2 as HYPK-interacting partner.**
(PDF)

Figure S3 **MALDI-MS identification of CALR as HYPK-interacting partner.**
(PDF)

Figure S4 **MALDI-MS identification of NME2 as HYPK-interacting partner.**
(PDF)

Figure S5 **MALDI-MS identification of PGAM1 as HYPK-interacting partner.**
(PDF)

Figure S6 **Validation of interaction of HTT interacting proteins with HYPK by co-immunoprecipitation and confocal imaging studies.** Validation of interaction between HYPK with HSPB1 (***A***), HSF1 (***B***), DNAJB3 (***C***), MLF1 (***D***), RELA (***E***), TP53 (***F***) and HIP1 (***G***) by co-IP and confocal imaging. Excepting for RELA (panel E), all the co-localization experiments were carried out in Neuro2A cells co-transfected with HYPK and these constructs fused with GFP or DsRed. Immunocytochemistry was performed in Neuro2A cells with anti-HYPK and anti-RELA antibodies. In all the cases, R^2^ values were analyzed for extent of co-localization (Table 2). In the co-IP experiments, anti-HYPK antibody was used to precipitate endogenous HYPK-bound protein and the western blot was probed with anti-HSPB1, RELA, TP53 or HIP1 antibodies. In case of TP53, the interaction was validated by antibody swapping. In the other cases, endogenous HYPK pull-down complexes were probed with either anti-GFP (in case of HSF1, DNAJB3 and MLF1) or anti-DsRed (for HIP1) antibodies.
(PDF)

Table S1 **Details of used antibodies.**
(PDF)

Table S2 **Primer sequences and constructs used in this study.** Details of cloning of HYPK-interacting protein-coding genes in mammalian expression vectors & HYPK antisense strand in pU61 RNA/Hygro vector and mouse *HYPK* expression primers are provided in Table S2A. All the constructs used in this study and their sources are detailed in Table S2B.
(PDF)

Table S3 **Details of MALDI analysis for the identification of HYPK-interacting proteins.**
(PDF)

Table S4 **Involvement of 49 HYPK-interacting partners in various Molecular Functions, Biological Processes and Pathways.** Enrichment of 47 Molecular Functions (MF) as observed with 49 HYPK-interacting partners is enlisted in Table S4A. The enrichment of 135 Biological Processes (BP) with the 49 HYPK-interacting proteins is provided in Table S4B. Enrichment of 16 different pathways as observed from KEGG pathway analysis and Reactome database analysis are detailed in Table S4C and Table S4D.
(PDF)

Table S5 **HYPK-interacting partners: sub-cellular localization and expression in brain.**
(PDF)

Table S6 **Mean values and standard deviations for growth curve calculation of Neuro2A and ST** ***Hdh^Q7^*** **/** ***Hdh^Q7^*** **cells with and without HYPK.**
(PDF)

## References

[1] Faber PW, Barnes GT, Srinidhi J, Chen J, Gusella JF, et al (1998) Huntingtin interacts with a family of WW domain proteins. Hum Mol Genet 7: 1463–1474. 9700202 10.1093/hmg/7.9.1463

[2] Raychaudhuri S, Sinha M, Mukhopadhyay D, Bhattacharyya NP (2008) HYPK, a Huntingtin interacting protein, reduces aggregates and apoptosis induced by N-terminal Huntingtin with 40 glutamines in Neuro2a cells and exhibits chaperone-like activity. Hum Mol Genet 17: 240–255. 17947297 10.1093/hmg/ddm301

[3] Raychaudhuri S, Majumder P, Sarkar S, Giri K, Mukhopadhyay D, et al (2008) Huntingtin interacting protein HYPK is intrinsically unstructured. Proteins 71: 1686–1698. 18076027 10.1002/prot.21856

[4] Arnesen T, Starheim KK, Van Damme P, Evjenth R, Dinh H, et al (2010) The chaperone-like protein HYPK acts together with NatA in cotranslational N-terminal acetylation and prevention of Huntingtin aggregation. Mol Cell Biol 30: 1898–1909. 20154145 10.1128/MCB.01199-09 PMC2849469

[5] Otto H, Conz C, Maier P, Wolfle T, Suzuki CK, et al (2005) The chaperones MPP11 and Hsp70L1 form the mammalian ribosome-associated complex. Proc Natl Acad Sci U S A 102: 10064–10069. 16002468 10.1073/pnas.0504400102 PMC1177401

[6] Stelzl U, Worm U, Lalowski M, Haenig C, Brembeck FH, et al (2005) A human protein-protein interaction network: a resource for annotating the proteome. Cell 122: 957–968. 16169070 10.1016/j.cell.2005.08.029

[7] Wacker JL, Zareie MH, Fong H, Sarikaya M, Muchowski PJ (2004) Hsp70 and Hsp40 attenuate formation of spherical and annular polyglutamine oligomers by partitioning monomer. Nat Struct Mol Biol 11: 1215–1222. 15543156 10.1038/nsmb860

[8] Barabasi AL, Gulbahce N, Loscalzo J (2011) Network medicine: a network-based approach to human disease. Nat Rev Genet 12: 56–68. 21164525 10.1038/nrg2918 PMC3140052

[9] Hegyi H, Schad E, Tompa P (2007) Structural disorder promotes assembly of protein complexes. BMC Struct Biol 7: 65. 17922903 10.1186/1472-6807-7-65 PMC2194777

[10] Patil A, Nakamura H (2006) Disordered domains and high surface charge confer hubs with the ability to interact with multiple proteins in interaction networks. FEBS Lett 580: 2041–2045. 16542654 10.1016/j.febslet.2006.03.003

[11] Tompa P (2005) The interplay between structure and function in intrinsically unstructured proteins. FEBS Lett 579: 3346–3354. 15943980 10.1016/j.febslet.2005.03.072

[12] Xie H, Vucetic S, Iakoucheva LM, Oldfield CJ, Dunker AK, et al (2007) Functional anthology of intrinsic disorder. 1. Biological processes and functions of proteins with long disordered regions. J Proteome Res 6: 1882–1898. 17391014 10.1021/pr060392u PMC2543138

[13] Raychaudhuri S, Dey S, Bhattacharyya NP, Mukhopadhyay D (2009) The role of intrinsically unstructured proteins in neurodegenerative diseases. PLoS One 4: e5566. 19440375 10.1371/journal.pone.0005566 PMC2679209

[14] Trettel F, Rigamonti D, Hilditch-Maguire P, Wheeler VC, Sharp AH, et al (2000) Dominant phenotypes produced by the HD mutation in STHdh(Q111) striatal cells. Hum Mol Genet 9: 2799–2809. 11092756 10.1093/hmg/9.19.2799

[15] Ghose J, Sinha M, Das E, Jana NR, Bhattacharyya NP (2011) Regulation of miR-146a by RelA/NFkB and p53 in STHdh(Q111)/Hdh(Q111) cells, a cell model of Huntington’s disease. PLoS One 6: e23837. 21887328 10.1371/journal.pone.0023837 PMC3162608

[16] Majumder P, Raychaudhuri S, Chattopadhyay B, Bhattacharyya NP (2007) Increased caspase-2, calpain activations and decreased mitochondrial complex II activity in cells expressing exogenous huntingtin exon 1 containing CAG repeat in the pathogenic range. Cell Mol Neurobiol 27: 1127–1145. 17902043 10.1007/s10571-007-9220-7 PMC11517176

[17] Bhattacharya D, Saha S, Basu S, Chakravarty S, Chakravarty A, et al (2010) Differential regulation of redox proteins and chaperones in HbEbeta-thalassemia erythrocyte proteome. Proteomics Clin Appl 4: 480–488. 21137065 10.1002/prca.200900073

[18] Carmona-Saez P, Chagoyen M, Tirado F, Carazo JM, Pascual-Montano A (2007) GENECODIS: a web-based tool for finding significant concurrent annotations in gene lists. Genome Biol 8: R3. 17204154 10.1186/gb-2007-8-1-r3 PMC1839127

[19] Nogales-Cadenas R, Carmona-Saez P, Vazquez M, Vicente C, Yang X, et al (2009) GeneCodis: interpreting gene lists through enrichment analysis and integration of diverse biological information. Nucleic Acids Res 37: W317–322. 19465387 10.1093/nar/gkp416 PMC2703901

[20] Croft D, O’Kelly G, Wu G, Haw R, Gillespie M, et al (2011) Reactome: a database of reactions, pathways and biological processes. Nucleic Acids Res 39: D691–697. 21067998 10.1093/nar/gkq1018 PMC3013646

[21] Lane L, Argoud-Puy G, Britan A, Cusin I, Duek PD, et al (2012) neXtProt: a knowledge platform for human proteins. Nucleic Acids Res 40: D76–83. 22139911 10.1093/nar/gkr1179 PMC3245017

[22] Novoselova TV, Margulis BA, Novoselov SS, Sapozhnikov AM, van der Spuy J, et al (2005) Treatment with extracellular HSP70/HSC70 protein can reduce polyglutamine toxicity and aggregation. J Neurochem 94: 597–606. 15992387 10.1111/j.1471-4159.2005.03119.x

[23] Mitsui K, Nakayama H, Akagi T, Nekooki M, Ohtawa K, et al (2002) Purification of polyglutamine aggregates and identification of elongation factor-1alpha and heat shock protein 84 as aggregate-interacting proteins. J Neurosci 22: 9267–9277. 12417652 10.1523/JNEUROSCI.22-21-09267.2002 PMC6758042

[24] Bao J, Sharp AH, Wagster MV, Becher M, Schilling G, et al (1996) Expansion of polyglutamine repeat in huntingtin leads to abnormal protein interactions involving calmodulin. Proc Natl Acad Sci U S A 93: 5037–5042. 8643525 10.1073/pnas.93.10.5037 PMC39402

[25] Bhattacharyya NP, Datta M, Banerjee M, Das S, Mukhopadhya S (2011) Huntingtin Interacting Proteins:Involvement in Diverse Molecular Functions, Biological Processes and Pathways. Nova Science Publishers. 39–59.

[26] Garcia-Mata R, Gao YS, Sztul E (2002) Hassles with taking out the garbage: aggravating aggresomes. Traffic 3: 388–396. 12010457 10.1034/j.1600-0854.2002.30602.x

[27] Bharadwaj S, Ali A, Ovsenek N (1999) Multiple components of the HSP90 chaperone complex function in regulation of heat shock factor 1 In vivo. Mol Cell Biol 19: 8033–8041. 10567529 10.1128/mcb.19.12.8033 PMC84888

[28] Fujimoto M, Takaki E, Hayashi T, Kitaura Y, Tanaka Y, et al (2005) Active HSF1 significantly suppresses polyglutamine aggregate formation in cellular and mouse models. J Biol Chem 280: 34908–34916. 16051598 10.1074/jbc.M506288200

[29] Hartwell LH, Hopfield JJ, Leibler S, Murray AW (1999) From molecular to modular cell biology. Nature 402: C47–52. 10591225 10.1038/35011540

[30] Frydman J, Nimmesgern E, Ohtsuka K, Hartl FU (1994) Folding of nascent polypeptide chains in a high molecular mass assembly with molecular chaperones. Nature 370: 111–117. 8022479 10.1038/370111a0

[31] Schneider C, Sepp-Lorenzino L, Nimmesgern E, Ouerfelli O, Danishefsky S, et al (1996) Pharmacologic shifting of a balance between protein refolding and degradation mediated by Hsp90. Proc Natl Acad Sci U S A 93: 14536–14541. 8962087 10.1073/pnas.93.25.14536 PMC26168

[32] Wu SY, Leu YL, Chang YL, Wu TS, Kuo PC, et al (2012) Physalin f induces cell apoptosis in human renal carcinoma cells by targeting NF-kappaB and generating reactive oxygen species. PLoS One 7: e40727. 22815798 10.1371/journal.pone.0040727 PMC3398016

[33] Imarisio S, Carmichael J, Korolchuk V, Chen CW, Saiki S, et al (2008) Huntington’s disease: from pathology and genetics to potential therapies. Biochem J 412: 191–209. 18466116 10.1042/BJ20071619

[34] Ross CA, Tabrizi SJ (2011) Huntington’s disease: from molecular pathogenesis to clinical treatment. Lancet Neurol 10: 83–98. 21163446 10.1016/S1474-4422(10)70245-3

[35] Helmbrecht K, Zeise E, Rensing L (2000) Chaperones in cell cycle regulation and mitogenic signal transduction: a review. Cell Prolif 33: 341–365. 11101008 10.1046/j.1365-2184.2000.00189.x PMC6496586

[36] Jolly C, Morimoto RI (2000) Role of the heat shock response and molecular chaperones in oncogenesis and cell death. J Natl Cancer Inst 92: 1564–1572. 11018092 10.1093/jnci/92.19.1564

[37] Godin JD, Colombo K, Molina-Calavita M, Keryer G, Zala D, et al (2010) Huntingtin is required for mitotic spindle orientation and mammalian neurogenesis. Neuron 67: 392–406. 20696378 10.1016/j.neuron.2010.06.027

[38] Abaza A, Soleilhac JM, Westendorf J, Piel M, Crevel I, et al (2003) M phase phosphoprotein 1 is a human plus-end-directed kinesin-related protein required for cytokinesis. J Biol Chem 278: 27844–27852. 12740395 10.1074/jbc.M304522200 PMC2652640

[39] Nasir J, Floresco SB, O’Kusky JR, Diewert VM, Richman JM, et al (1995) Targeted disruption of the Huntington’s disease gene results in embryonic lethality and behavioral and morphological changes in heterozygotes. Cell 81: 811–823. 7774020 10.1016/0092-8674(95)90542-1

[40] Zeitlin S, Liu JP, Chapman DL, Papaioannou VE, Efstratiadis A (1995) Increased apoptosis and early embryonic lethality in mice nullizygous for the Huntington’s disease gene homologue. Nat Genet 11: 155–163. 7550343 10.1038/ng1095-155

