# Supplementary figures and images for: Identification of HYPK-Interacting Proteins Reveals Involvement of HYPK in Regulating Cell Growth, Cell Cycle, Unfolded Protein Response and Cell Death
Source: PLoS One. 2012 Dec 10;7(12):e51415. doi: 10.1371/journal.pone.0051415 (PMC3525516; doi:10.1371/journal.pone.0051415)

Supplementary Figure S1: MOWSE score distribution (A), parental MS (B) and MS/MS (C) spectra for HSPA8

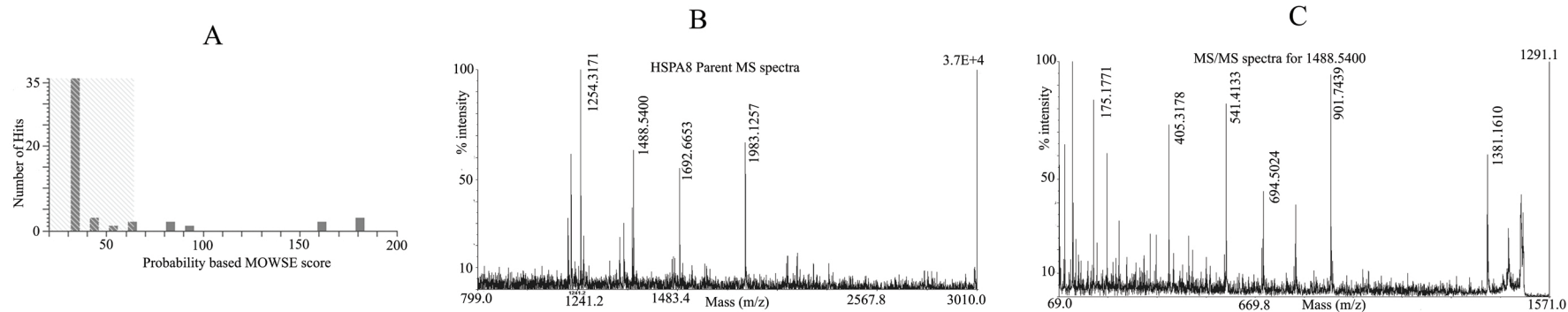

Supplement: Figure S1 — MALDI-MS identification of HSPA8 as HYPK-interacting partner. (PDF) [file pone.0051415.s001.pdf]

Supplementary Figure S2: MOWSE score distribution (A), parental MS (B) and MS/MS (C) spectra for LMNB2

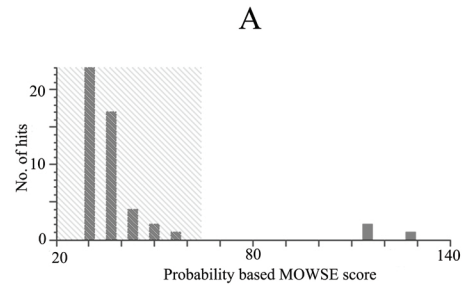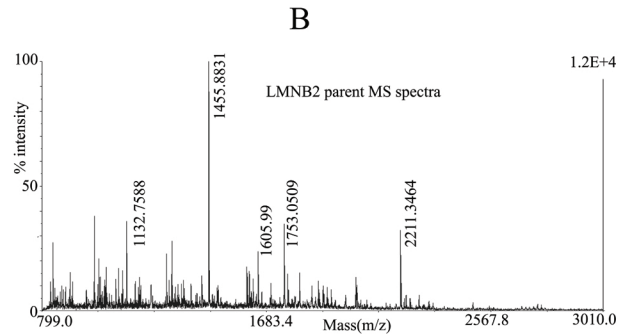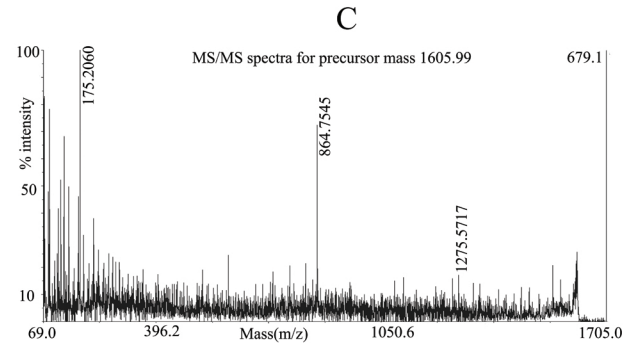

Supplement: Figure S2 — MALDI-MS identification of LMNB2 as HYPK-interacting partner. (PDF) [file pone.0051415.s002.pdf]

Supplementary Figure S3: MOWSE score distribution (A), parental MS (B) and MS/MS (C) spectra for CALR

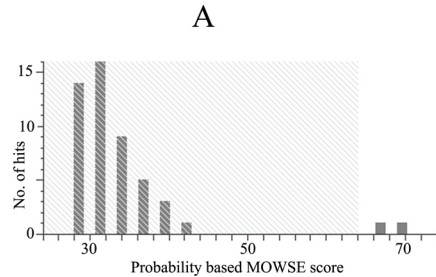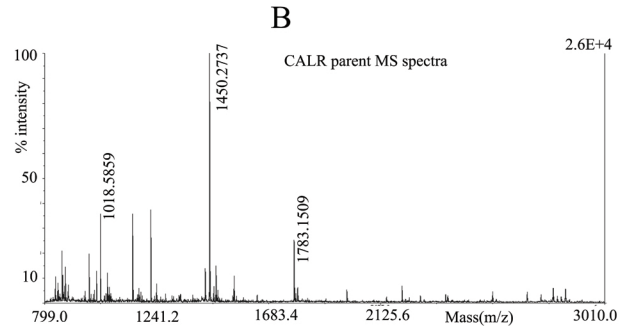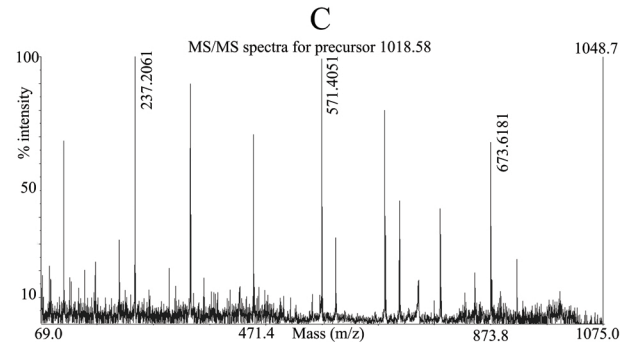

Supplement: Figure S3 — MALDI-MS identification of CALR as HYPK-interacting partner. (PDF) [file pone.0051415.s003.pdf]

Supplementary Figure S4: MOWSE score distribution (A), parental MS (B) and MS/MS (C) spectra for NME2

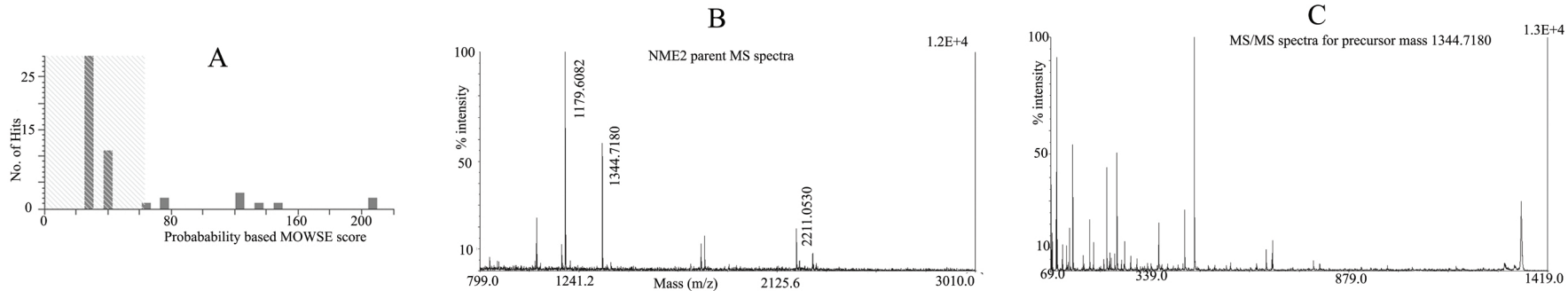

Supplement: Figure S4 — MALDI-MS identification of NME2 as HYPK-interacting partner. (PDF) [file pone.0051415.s004.pdf]

Supplementary Figure S5: MOWSE score distribution (A), parental MS (B) and MS/MS (C) spectra for PGAM1

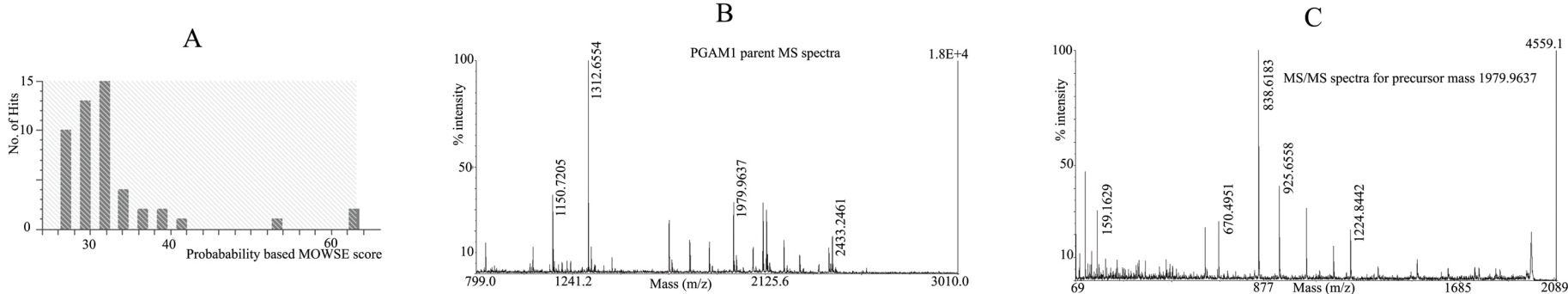

Supplement: Figure S5 — MALDI-MS identification of PGAM1 as HYPK-interacting partner. (PDF) [file pone.0051415.s005.pdf]
