# Supplementary material for: Identification of HYPK-Interacting Proteins Reveals Involvement of HYPK in Regulating Cell Growth, Cell Cycle, Unfolded Protein Response and Cell Death
Source: PLoS One. 2012 Dec 10;7(12):e51415. doi: 10.1371/journal.pone.0051415 (PMC3525516; doi:10.1371/journal.pone.0051415)

Supplementary Figure S6: Validation of interaction of HTT interacting proteins with HYPK by co-immunoprecipitation and confocal imaging studies

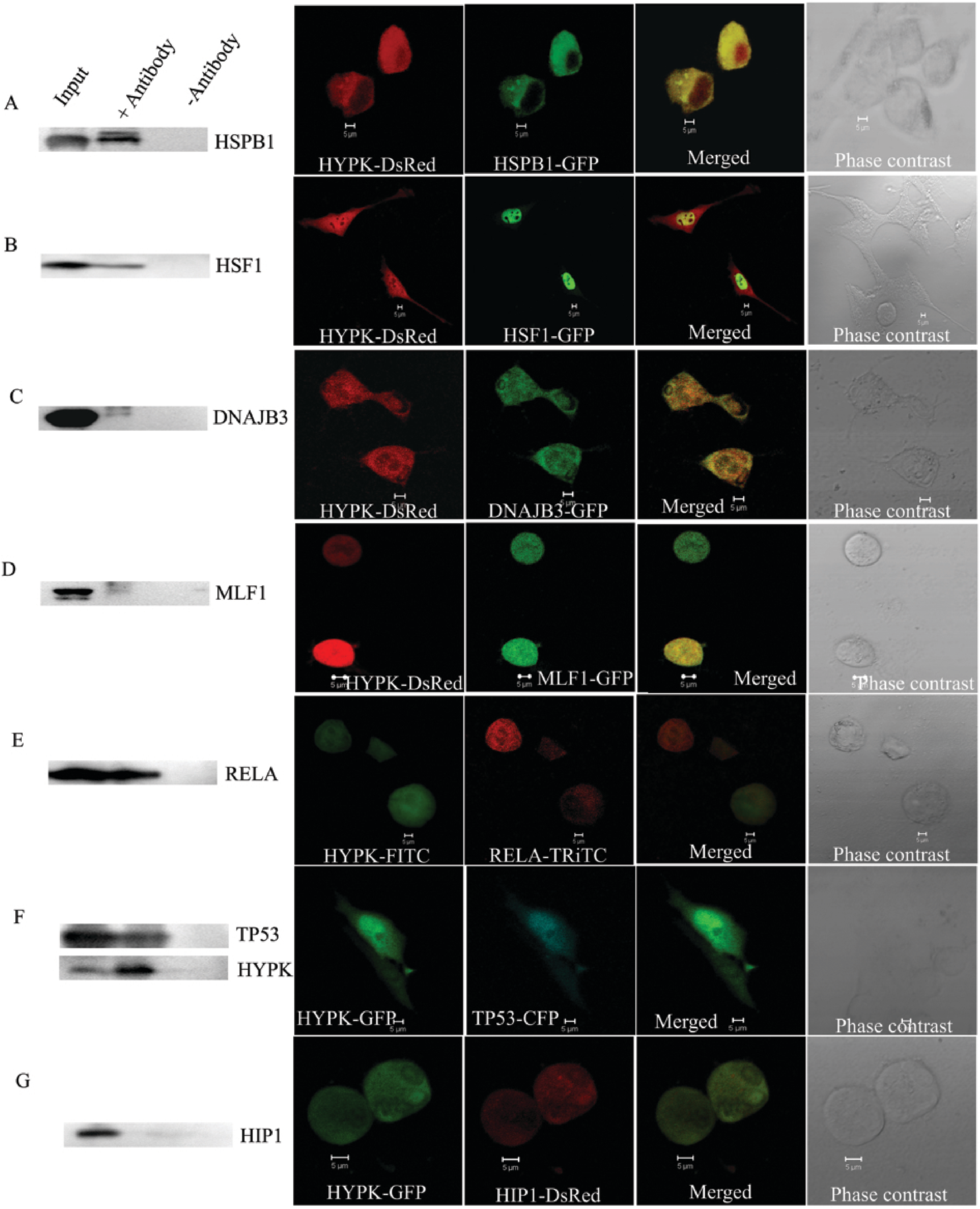

Supplement: Figure S6 — Validation of interaction of HTT interacting proteins with HYPK by co-immunoprecipitation and confocal imaging studies. Validation of interaction between HYPK with HSPB1 (A), HSF1 (B), DNAJB3 (C), MLF1 (D), RELA (E), TP53 (F) and HIP1 (G) by co-IP and confocal imaging. Excepting for RELA (panel E), all the co-localization experiments were carried out in Neuro2A cells co-transfected with HYPK and these constructs fused with GFP or DsRed. Immunocytochemistry was performed in Neuro2A cells with anti-HYPK and anti-RELA antibodies. In all the cases, R2 values were analyzed for extent of co-localization (Table 2). In the co-IP experiments, anti-HYPK antibody was used to precipitate endogenous HYPK-bound protein and the western blot was probed with anti-HSPB1, RELA, TP53 or HIP1 antibodies. In case of TP53, the interaction was validated by antibody swapping. In the other cases, endogenous HYPK pull-down complexes were probed with either anti-GFP (in case of HSF1, DNAJB3 and MLF1) or anti-DsRed (for HIP1) antibodies. (PDF) [file pone.0051415.s006.pdf]
