# Supplementary material for: Identification of HYPK-Interacting Proteins Reveals Involvement of HYPK in Regulating Cell Growth, Cell Cycle, Unfolded Protein Response and Cell Death
Source: PLoS One. 2012 Dec 10;7(12):e51415. doi: 10.1371/journal.pone.0051415 (PMC3525516; doi:10.1371/journal.pone.0051415)
Supplement: Table S1 — Details of used antibodies. (PDF) [file pone.0051415.s007.pdf]

Supplementary Table S1:

Details of used antibodies

Antibodies used	Source	Used in
Mouse anti-EF1α	Upstate, Cat no. 05-235	Co-immunoprecipitation and Native-PAGE
Mouse anti-Hsp70	Abcam monoclonal, Cat. no. ab6535	Co-immunoprecipitation and Native-PAGE
Rabbit anti-Hsp27	Abcam polyclonal, Cat. no. ab12351	Co-immunoprecipitation
Rabbit anti-DsRedC1	Clontech polyclonal, Cat. no. 632496	Co-immunoprecipitation
Mouse anti-NFκβ against the p65 subunit	Millipore monoclonal, Cat. no. MAB3026	Co-immunoprecipitation, Immunocytochemistry and Native-PAGE
Mouse anti-p53	Imgenex monoclonal, Cat. no. IMG-80061	Co-immunoprecipitation and Native-PAGE
Mouse anti-GFPC1	Clontech monoclonal, Cat. No. 632375	Co-immunoprecipitation
Mouse anti-HIP1	Novus Biologicals, Cat no. NB300-204, 1B11	Co-immunoprecipitation
Rabbit anti-HYPK	Anti-HYPK polyclonal antibody was custom made by providing purified HYPK protein to Imgenex Biotech Pvt. Ltd, India, polyclonal, CP 40 07	Co-immunoprecipitation, Native PAGE, Immunocytochemistry
Mouse anti-LaminB2	Abcam monoclonal, Cat. no. ab8983	Co-immunoprecipitation, Native PAGE
Mouse anti-HTT (clone 1HU-4C8)	Millipore monoclonal, Cat. no. MAB2166)	Co-immunoprecipitation, Native PAGE

Anti-mouse secondary antibody (conjugated with TRiTC, excitation maxima 541 nm)	Bangalore Genei, Cat no. 106008	Immunocytochemistry
Anti-rabbit secondary antibody (conjugated with FITC, excitation maxima 494 nm)	Bangalore Genei, India, Cat no. 10547	Immunocytochemistry
Goat Anti-Mouse IgG HRP conjugate	Bangalore Genei, India, Cat no. 105502	Secondary antibody in Western Blotting
Goat Anti-Rabbit IgG HRP conjugate	Bangalore Genei, India, Cat no. 105499	Secondary antibody in Western Blotting
