# Supplementary material for: Identification of HYPK-Interacting Proteins Reveals Involvement of HYPK in Regulating Cell Growth, Cell Cycle, Unfolded Protein Response and Cell Death
Source: PLoS One. 2012 Dec 10;7(12):e51415. doi: 10.1371/journal.pone.0051415 (PMC3525516; doi:10.1371/journal.pone.0051415)
Supplement: Table S2 — Primer sequences and constructs used in this study. Details of cloning of HYPK-interacting protein-coding genes in mammalian expression vectors & HYPK antisense strand in pU61 RNA/Hygro vector and mouse HYPK expression primers are provided in Table S2A. All the constructs used in this study and their sources are detailed in Table S2B. (PDF) [file pone.0051415.s008.pdf]

Supplementary Table S2: ***Primer sequences and constructs used in this study***

Supplementary Table S2A: Cloning of HYPK-interacting protein-coding genes in mammalian expression vectors, HYPK antisense strand in pU6.1 RNA/Hygro vector & Mus musculus HYPK expression primers

GENE	VECTOR	SEQUENCE OF FORWARD PRIMER	RESTRICTION ENZYME SITE IN FORWARD ADAPTOR SEQUENCE	SEQUENCE OF REVERSE PRIMER	RESTRICTION ENZYME SITE IN REVERSE ADAPTOR SEQUENCE
HSPA8	DsRed-C1	5'-ACGCGTCGACGTCAAGGGACCTGCAGTTGGTA-3'	SalI	5'-CGGGATCCTTAATCAACCTCTTCAATGGTG-3'	BamHI
CALM1	DsRed-C1	5'-ACGCGTCGACGTATGGCTGATCAGCTGACTGAAGAG-3'	SalI	5'-CGGGATCCTCATTTTGCAGTCATCATCTGT-3'	BamHI
LMNB2	DsRed-C1	5'-ACGCGTCGACATGGCCACGCCGCTG-3'	SalI	5'-CGGGATCCTCACATCACGTAGCAGCCTCT-3'	BamHI
VIM	DsRed-C1	5'-ACGCGTCGACGTGTCCACCAGGTCCGTGTC -3'	SalI	5'-CGGGATCCTTCTCAGCATCACGATGACC-3'	BamHI
Antisense HYPK	pU6.1 RNA/Hygro	5'-CGGGATCCTAGGGCAATAAGAGCCTCCA-3'	BamHI	5'-CCCAAGCTTTTTTTGGAGATCTCTCGAGCAGCAG-3'	HindIII
**EXPRESSION PRIMERS**					
Mouse HYPK expression primer	N/A	5'-GCGGTGAGATCGAAATGG-3'	N/A	5'-TCAGTTGGTTAGGGCAATAAGA-3'	N/A

Supplementary Table S2B: Summary & sources of the constructs used in this study

GENE CLONED	VECTOR(S)	DESIGNATION	SOURCES
HSPA8	pECFP-C1, pDsRed-C1	HSPA8-CFP, HSPA8-DsRed	Present Study
CALM1	pEGFP-C1, pDsRed-C1	CALM1-GFP, CALM1-DsRed	Present Study
LMNB2	pDsRed-C1	LMNB2-DsRed	Present Study
VIM	pDsRed-C1	VIM-DsRed	Present Study
HYPK	pEGFP-C1, pDsRed-C1	HYPK-GFP, HYPK-DsRed	Swasti Raychaudhuri et al., Human Molecular Genetics, 2008, 17(2): 240–255
EEF1A1	pDsRed-C1, pEGFP-C1	EEF1A1-DsRed, EEF1A1-GFP	Swasti Raychaudhuri, Srijit Das and N.P. Bhattacharyya, Unpublished data
HSPA1A	pEGFP-C1	HSPA1A-GFP	Swasti Raychaudhuri and N.P. Bhattacharyya, Unpublished data
HSPB1	pEGFP-C1	HSPB1-GFP	Kindly gifted by Dr. Nihar Ranjan Jana, National Brain Research Centre, India
DNAJB3	pEGFP-C1	DNAJB3-GFP	Manisha Banerjee and N.P. Bhattacharyya, Unpublished data
MLF1	pEGFP-C1	MLF1-GFP	Manisha Banerjee and N.P. Bhattacharyya, Unpublished data
MLF2	pEGFP-C1	MLF2-GFP	Manisha Banerjee and N.P. Bhattacharyya, Unpublished data
HIP1	pDsRed-C1	HIP1-DsRed	Manisha Banerjee and N.P. Bhattacharyya, Unpublished data
TP53	pECFP-C1	TP53-CFP	Jayeeta Ghose et al., PLoS One. 2011;6(8):e23837
HSF1	pEGFP-C1	HSF1-GFP	Srijit Das and N.P. Bhattacharyya, Unpublished data
Antisense HYPK	pU6.1-RNA/Hygro	HYPK-U61	Present Study
