# Supplementary material for: Identification of HYPK-Interacting Proteins Reveals Involvement of HYPK in Regulating Cell Growth, Cell Cycle, Unfolded Protein Response and Cell Death
Source: PLoS One. 2012 Dec 10;7(12):e51415. doi: 10.1371/journal.pone.0051415 (PMC3525516; doi:10.1371/journal.pone.0051415)
Supplement: Table S3 — Details of MALDI analysis for the identification of HYPK-interacting proteins. (PDF) [file pone.0051415.s009.pdf]

<u>Supplementary Table S3:</u> Details of MALDI analysis for the identification of HYPK interacting proteins

HUGO name of interacting partner	Score	Mol.Mass (kDa)	pI	Peptides matched	%Sequence coverage
HSPA8	181	64.633	5.36	43	54
LMNB2	56	43.34	9.17	25	59
CALM1	67	17.15	4.06	10	72
EEF1A1	63	50.09	9.1	11	20
LBR	69	10	10.32	5	56
ARCN1	66	57.17	5.89	34	46
TPI1	40	26.93	8.21	24	60
LENG8	64	88.1	9.24	25	27
SDCCAG1	40	23.82	7.66	28	52
IKBIP	47	39.29	9.21	17	30
CDH11	53	66.72	4.75	18	39
CALR	70	46.89	4.3	17	47
CEP290	71	29.02	5.76	59	42
HSP90AB1	139	83.21	4.97	32	34
MYOM3	64	131.39	5.49	20	16
ZNF462	67	119.53	6.07	39	61
ZNF516	57	124.21	9.02	33	28
ZNF100	68	63	9.14	23	45
KIF20B	62	210.43	5.55	48	22
PGAM1	63	28.79	6.67	11	37
NME2	276	17.29	8.52	26	70
GLUD1	133	61.7	7.66	45	49
GLUD2	103	61.74	8.63	36	44
N4BP1	63	100.97	5.24	25	46
ATP6V0A4	59	96.35	6.02	25	28
SRRT	52	100.14	5.61	20	29
PPP6R2	62	23.03	4.02	14	36
MAVS	61	56.49	4.36	4	36
