# Supplementary material for: Identification of HYPK-Interacting Proteins Reveals Involvement of HYPK in Regulating Cell Growth, Cell Cycle, Unfolded Protein Response and Cell Death
Source: PLoS One. 2012 Dec 10;7(12):e51415. doi: 10.1371/journal.pone.0051415 (PMC3525516; doi:10.1371/journal.pone.0051415)
Supplement: Table S4 — Involvement of 49 HYPK-interacting partners in various Molecular Functions, Biological Processes and Pathways. Enrichment of 47 Molecular Functions (MF) as observed with 49 HYPK-interacting partners is enlisted in Table S4A. The enrichment of 135 Biological Processes (BP) with the 49 HYPK-interacting proteins is provided in Table S4B. Enrichment of 16 different pathways as observed from KEGG pathway analysis and Reactome database analysis are detailed in Table S4C and Table S4D. (PDF) [file pone.0051415.s010.pdf]

<u>Supplementary Table S4:</u> ***Involvement of 49 HYPK-interacting partners in various Molecular Functions, Biological Processes and Pathways***

Supplementary Table S4A: Enrichment of 47 Molecular Functions (MF) with 49 HYPK-interacting proteins

GO terms (MF)	Annotations	Genes	S	TS	Hyp	Hyp_c	Genes
GO:0005515	protein binding (MF)	29 genes	29(45)	6618(29095)	2.67E-09	2.35E-07	ARCN1,TPI1,CHD3,RELA,HSF1,ATP6V0A4,RBPMS,TP53,TH1L,HSP90AB1,HTT,MDFI,PSME3,QKI,HSPA8,GLUD1,RHOXF2,MLF1,VIM,CDH11,LBR,LENG8,SRRT,MLF2,CEP290,EEF1A1,CALR,IKBIP,HSPB1
GO:0004352	glutamate dehydrogenase activity (MF)	2 genes	2(45)	2(29095)	2.34E-06	5.15E-05	GLUD2,GLUD1
GO:0004353	glutamate dehydrogenase [NAD(P)+] activity (MF)	2 genes	2(45)	2(29095)	2.34E-06	5.15E-05	GLUD2,GLUD1
GO:0070728	leucine binding (MF)	2 genes	2(45)	2(29095)	2.34E-06	5.15E-05	GLUD2,GLUD1
GO:0051082	unfolded protein binding (MF)	4 genes	4(45)	111(29095)	2.65E-05	0.0004663	DNAJC2,HSP90AB1,HSPA8,CALR
GO:0003677	DNA binding (MF)	10 genes	10(45)	1454(29095)	6.04E-05	0.0008863	ZNF462,CHD3,RELA,TP53,DNAJC2,MLF1,LBR,ZNF516,ZNF100,CALR
GO:0043531	ADP binding (MF)	2 genes	2(45)	19(29095)	0.0003933	0.0049448	GLUD2,GLUD1
GO:0019901	protein kinase binding (MF)	3 genes	3(45)	112(29095)	0.0007004	0.0077044	PGAM1,RELA,VIM
GO:0051087	chaperone binding (MF)	2 genes	2(45)	29(29095)	0.0009248	0.009042	TP53,CALR
GO:0004807	triose-phosphate isomerase activity (MF)	1 gene	1(45)	1(29095)	0.0015467	0.0104697	TPI1
GO:0050809	diazepam binding (MF)	1 gene	1(45)	1(29095)	0.0015467	0.0104697	HTT
GO:0051011	microtubule minus-end binding (MF)	1 gene	1(45)	1(29095)	0.0015467	0.0104697	CEP290
GO:0051183	vitamin transporter activity (MF)	1 gene	1(45)	1(29095)	0.0015467	0.0104697	GC
GO:0042802	identical protein binding (MF)	4 genes	4(45)	336(29095)	0.0017925	0.0112669	RELA,PSME3,GLUD1,HSPB1
GO:0050613	delta14-sterol reductase activity (MF)	1 gene	1(45)	2(29095)	0.003091	0.0181337	LBR
GO:0010843	promoter binding (MF)	2 genes	2(45)	77(29095)	0.0063581	0.0223803	RELA,TP53
GO:0016563	transcription activator activity (MF)	3 genes	3(45)	221(29095)	0.0048505	0.0224653	RELA,TP53,CEP290
GO:00	DNA strand	1 gene	1(45)	4(29095)	0.006	0.0226329	TP53

00739	annealing activity (MF)				1726		
GO:0004082	bisphosphoglycerate mutase activity (MF)	1 gene	1(45)	4(29095)	0.0061726	0.0226329	PGAM1
GO:0004083	2,3-bisphospho-D-glycerate 2-phosphohydrolase activity (MF)	1 gene	1(45)	4(29095)	0.0061726	0.0226329	PGAM1
GO:0004619	phosphoglycerate mutase activity (MF)	1 gene	1(45)	4(29095)	0.0061726	0.0226329	PGAM1
GO:0001849	complement component C1q binding (MF)	1 gene	1(45)	3(29095)	0.004633	0.02265	CALR
GO:0030911	TPR domain binding (MF)	1 gene	1(45)	3(29095)	0.004633	0.02265	HSP90AB1
GO:0070215	MDM2 binding (MF)	1 gene	1(45)	3(29095)	0.004633	0.02265	PSME3
GO:0005524	ATP binding (MF)	7 genes	7(45)	1467(29095)	0.0069006	0.0233558	CHD3,TP53,HSP90AB1,HSPA8,GLUD1,KIF20B,HSPA14
GO:0005499	vitamin D binding (MF)	1 gene	1(45)	5(29095)	0.0077099	0.0233957	GC
GO:0008538	proteasome activator activity (MF)	1 gene	1(45)	5(29095)	0.0077099	0.0233957	PSME3
GO:0030235	nitric-oxide synthase regulator activity (MF)	1 gene	1(45)	5(29095)	0.0077099	0.0233957	HSP90AB1
GO:0047485	protein N-terminus binding (MF)	2 genes	2(45)	73(29095)	0.0057329	0.0252248	RELA,TP53
GO:0005521	lamin binding (MF)	1 gene	1(45)	6(29095)	0.0092449	0.0254236	LBR
GO:0070087	chromo shadow domain binding (MF)	1 gene	1(45)	6(29095)	0.0092449	0.0254236	LBR
GO:0000166	nucleotide binding (MF)	8 genes	8(45)	1928(29095)	0.0087422	0.0256438	CHD3,RBPMS,HSP90AB1,HSPA8,GLUD1,KIF20B,HSPA14,EEF1A1
GO:0008553	hydrogen-exporting ATPase activity, phosphorylative mechanism (MF)	1 gene	1(45)	7(29095)	0.0107776	0.0287403	ATP6V0A4
GO:0016853	isomerase activity (MF)	2 genes	2(45)	106(29095)	0.0117546	0.0304237	PGAM1,TPI1
GO:0033613	transcription activator binding (MF)	1 gene	1(45)	8(29095)	0.012308	0.0309457	RELA
GO:0008143	poly(A) RNA binding (MF)	1 gene	1(45)	9(29095)	0.013836	0.0338213	RBPMS
GO:0042623	ATPase activity, coupled (MF)	1 gene	1(45)	10(29095)	0.0153617	0.0355745	HSPA8

GO:0070403	NAD binding (MF)	1 gene	1(45)	10(29095)	0.0153617	0.0355745	GLUD1
GO:0005525	GTP binding (MF)	3 genes	3(45)	365(29095)	0.0188377	0.0404321	GLUD2,GLUD1,EEF1A1
GO:0005246	calcium channel regulator activity (MF)	1 gene	1(45)	12(29095)	0.0184063	0.0404938	HTT
GO:0070491	transcription repressor binding (MF)	1 gene	1(45)	12(29095)	0.0184063	0.0404938	RELA
GO:0003682	chromatin binding (MF)	2 genes	2(45)	139(29095)	0.0196177	0.0411037	CHD3,TP53
GO:0051117	ATPase binding (MF)	1 gene	1(45)	14(29095)	0.0214416	0.0438805	ATP6V0A4
GO:0008134	transcription factor binding (MF)	2 genes	2(45)	153(29095)	0.0234629	0.0448855	RELA,TP53
GO:0042301	phosphate binding (MF)	1 gene	1(45)	15(29095)	0.0229558	0.0448914	RELA
GO:0051059	NF-kappaB binding (MF)	1 gene	1(45)	15(29095)	0.0229558	0.0448914	RELA

Supplementary Table S4B: Enrichment of 135 Biological Processes (BP) with 49 HYPK-interacting partners

Annotations	Items	Genes	S	TS	Hyp	Hyp_c	Genes
glutamate deamidation (BP)	GO:0019459	2 genes	2(45)	2(29095)	2.34E-06	0.0006	GLUD2,GLUD1
glutamate biosynthetic process (BP)	GO:0006537	2 genes	2(45)	5(29095)	2.33E-05	0.0019	GLUD2,GLUD1
cell cycle arrest (BP)	GO:0007050	4 genes	4(45)	106(29095)	2.21E-05	0.0027	TP53,MLF1,KIF20B,CALR
negative regulation of DNA binding (BP)	GO:0043392	2 genes	2(45)	9(29095)	8.36E-05	0.0034	ZNF462,MDFI
regulation of mitochondrial membrane permeability (BP)	GO:0046902	2 genes	2(45)	10(29095)	0.000104	0.0037	TP53,HTT
response to unfolded protein (BP)	GO:0006986	3 genes	3(45)	50(29095)	6.44E-05	0.0039	HSP90AB1,HSPA8,HSPB1
protein folding (BP)	GO:0006457	4 genes	4(45)	149(29095)	8.36E-05	0.0041	DNAJC2,HSP90AB1,HSPA8,CALR
embryonic development (BP)	GO:0009790	3 genes	3(45)	69(29095)	0.000169	0.0052	TPI1,HTT,MDFI
gastrulation (BP)	GO:0007369	2 genes	2(45)	15(29095)	0.000242	0.0059	TP53,HTT
response to X-ray (BP)	GO:0010165	2 genes	2(45)	15(29095)	0.000242	0.0059	TP53,IKBIP
cell aging (BP)	GO:0007569	2 genes	2(45)	27(29095)	0.000801	0.0164	TP53,HTT
regulation of apoptosis (BP)	GO:0042981	3 genes	3(45)	114(29095)	0.000737	0.0164	TP53,PSME3,CALR
citrulline metabolic process (BP)	GO:0000052	1 gene	1(45)	1(29095)	0.001547	0.0172	HTT
peptide antigen assembly with MHC class I protein complex (BP)	GO:0002502	1 gene	1(45)	1(29095)	0.001547	0.0172	CALR
positive regulation of Schwann cell differentiation (BP)	GO:0014040	1 gene	1(45)	1(29095)	0.001547	0.0172	RELA
lactate biosynthetic process from pyruvate (BP)	GO:0019244	1 gene	1(45)	1(29095)	0.001547	0.0172	HTT
olfactory lobe development (BP)	GO:0021988	1 gene	1(45)	1(29095)	0.001547	0.0172	HTT
neural plate formation (BP)	GO:0021990	1 gene	1(45)	1(29095)	0.001547	0.0172	HTT
regulation of pentose-phosphate shunt (BP)	GO:0043456	1 gene	1(45)	1(29095)	0.001547	0.0172	PGAM1
pronephros development (BP)	GO:0048793	1 gene	1(45)	1(29095)	0.001547	0.0172	CEP290
vitamin transport (BP)	GO:0051180	1 gene	1(45)	1(29095)	0.001547	0.0172	GC
cellular amino acid metabolic process (BP)	GO:0006520	2 genes	2(45)	40(29095)	0.001758	0.0187	GLUD2,GLUD1
regulation of cell cycle (BP)	GO:0051726	2 genes	2(45)	42(29095)	0.001936	0.0198	TP53,HSPA8
T cell proliferation during immune response (BP)	GO:0002309	1 gene	1(45)	2(29095)	0.003091	0.0205	TP53
glutamate catabolic process (BP)	GO:0006538	1 gene	1(45)	2(29095)	0.003091	0.0205	GLUD1

glyceraldehyde-3-phosphate metabolic process (BP)	GO:0019682	1 gene	1(45)	2(29095)	0.003091	0.0205	TPI1
negative regulation of steroid hormone receptor signaling pathway (BP)	GO:0033144	1 gene	1(45)	2(29095)	0.003091	0.0205	CALR
response to cobalamin (BP)	GO:0033590	1 gene	1(45)	2(29095)	0.003091	0.0205	RELA
long-chain fatty acid biosynthetic process (BP)	GO:0042759	1 gene	1(45)	2(29095)	0.003091	0.0205	QKI
intermediate filament-based process (BP)	GO:0045103	1 gene	1(45)	2(29095)	0.003091	0.0205	VIM
negative regulation of helicase activity (BP)	GO:0051097	1 gene	1(45)	2(29095)	0.003091	0.0205	TP53
regulation of interferon-gamma-mediated signaling pathway (BP)	GO:0060334	1 gene	1(45)	2(29095)	0.003091	0.0205	HSP90AB1
regulation of type I interferon-mediated signaling pathway (BP)	GO:0060338	1 gene	1(45)	2(29095)	0.003091	0.0205	HSP90AB1
interspecies interaction between organisms (BP)	GO:0044419	4 genes	4(45)	300(29095)	0.001183	0.0223	RELA,TP53,HSPA8,VIM
response to heat (BP)	GO:0009408	2 genes	2(45)	46(29095)	0.002318	0.0227	HSF1,HSPB1
cellular membrane organization (BP)	GO:0016044	2 genes	2(45)	58(29095)	0.003659	0.0236	ARCN1,HSPA8
anti-apoptosis (BP)	GO:0006916	3 genes	3(45)	182(29095)	0.002817	0.0256	RELA,HTT,HSPB1
ossification (BP)	GO:0001503	2 genes	2(45)	50(29095)	0.002733	0.0258	ATP6V0A4,CDH11
myeloid progenitor cell differentiation (BP)	GO:0002318	1 gene	1(45)	3(29095)	0.004633	0.0264	MLF1
response to tumor cell (BP)	GO:0002347	1 gene	1(45)	3(29095)	0.004633	0.0264	TP53
quinolinate biosynthetic process (BP)	GO:0019805	1 gene	1(45)	3(29095)	0.004633	0.0264	HTT
otic vesicle formation (BP)	GO:0030916	1 gene	1(45)	3(29095)	0.004633	0.0264	CEP290
sequestering of calcium ion (BP)	GO:0051208	1 gene	1(45)	3(29095)	0.004633	0.0264	CALR
negative regulation of transcription from RNA polymerase II promoter (BP)	GO:0000122	3 genes	3(45)	221(29095)	0.00485	0.027	TP53,MDFI,CALR
response to lipopolysaccharide (BP)	GO:0032496	2 genes	2(45)	76(29095)	0.006199	0.0281	RELA,HSF1
regulation of glycolysis (BP)	GO:0006110	1 gene	1(45)	4(29095)	0.006173	0.0285	PGAM1
embryonic development ending in birth or egg hatching (BP)	GO:0009792	1 gene	1(45)	4(29095)	0.006173	0.0285	TP53
dorsal/ventral axis specification (BP)	GO:0009950	1 gene	1(45)	4(29095)	0.006173	0.0285	MDFI
negative regulation of proteasomal ubiquitin-dependent protein catabolic process (BP)	GO:0032435	1 gene	1(45)	4(29095)	0.006173	0.0285	HSP90AB1
cellular response to glucose starvation (BP)	GO:0042149	1 gene	1(45)	4(29095)	0.006173	0.0285	TP53
L-glutamate import (BP)	GO:0051	1 gene	1(45)	4(29095)	0.006173	0.0285	HTT

	938						
nucleotide-binding oligomerization domain containing 2 signaling pathway (BP)	GO:0070431	1 gene	1(45)	4(29095)	0.006173	0.0285	RELA
M phase of mitotic cell cycle (BP)	GO:0000087	1 gene	1(45)	6(29095)	0.009245	0.0298	KIF20B
pentose-phosphate shunt (BP)	GO:0006098	1 gene	1(45)	6(29095)	0.009245	0.0298	TPI1
negative regulation of neuroblast proliferation (BP)	GO:0007406	1 gene	1(45)	6(29095)	0.009245	0.0298	TP53
striatum development (BP)	GO:0021756	1 gene	1(45)	6(29095)	0.009245	0.0298	HTT
DNA damage response, signal transduction by p53 class mediator (BP)	GO:0030330	1 gene	1(45)	6(29095)	0.009245	0.0298	TP53
G1/S DNA damage checkpoint (BP)	GO:0031571	1 gene	1(45)	6(29095)	0.009245	0.0298	TP53
vitamin D metabolic process (BP)	GO:0042359	1 gene	1(45)	6(29095)	0.009245	0.0298	GC
glucocorticoid receptor signaling pathway (BP)	GO:0042921	1 gene	1(45)	6(29095)	0.009245	0.0298	CALR
negative regulation of fibroblast proliferation (BP)	GO:0048147	1 gene	1(45)	6(29095)	0.009245	0.0298	TP53
negative regulation of retinoic acid receptor signaling pathway (BP)	GO:0048387	1 gene	1(45)	6(29095)	0.009245	0.0298	CALR
embryonic process involved in female pregnancy (BP)	GO:0060136	1 gene	1(45)	6(29095)	0.009245	0.0298	HSF1
B cell lineage commitment (BP)	GO:0002326	1 gene	1(45)	5(29095)	0.00771	0.03	TP53
T cell lineage commitment (BP)	GO:0002360	1 gene	1(45)	5(29095)	0.00771	0.03	TP53
axon cargo transport (BP)	GO:0008088	1 gene	1(45)	5(29095)	0.00771	0.03	HTT
protein maturation by protein folding (BP)	GO:0022417	1 gene	1(45)	5(29095)	0.00771	0.03	CALR
primary microRNA processing (BP)	GO:0031053	1 gene	1(45)	5(29095)	0.00771	0.03	SRRT
cellular response to UV (BP)	GO:0034644	1 gene	1(45)	5(29095)	0.00771	0.03	TP53
regulation of meiosis (BP)	GO:0040020	1 gene	1(45)	5(29095)	0.00771	0.03	CALR
paraxial mesoderm formation (BP)	GO:0048341	1 gene	1(45)	5(29095)	0.00771	0.03	HTT
iron ion homeostasis (BP)	GO:0055072	1 gene	1(45)	5(29095)	0.00771	0.03	HTT
regulation of transcription, DNA-dependent (BP)	GO:0006355	6 genes	6(45)	1063(29095)	0.005656	0.0308	CHD3,HSF1,TP53,RHOXF2,ZNF100,CALR
negative regulation of cell growth (BP)	GO:0030308	2 genes	2(45)	88(29095)	0.008229	0.031	TP53,DNAJC2
spermatogenesis (BP)	GO:0007283	3 genes	3(45)	267(29095)	0.008161	0.0312	HSF1,HTT,QKI
defense response (BP)	GO:0006952	2 genes	2(45)	74(29095)	0.005886	0.0314	HSF1,MLF2
G2 phase of mitotic cell	GO:0000	1 gene	1(45)	7(29095)	0.010778	0.0314	DNAJC2

cycle (BP)	085						
dopamine receptor signaling pathway (BP)	GO:0007 212	1 gene	1(45)	7(29095)	0.010778	0.0314	HTT
response to salt stress (BP)	GO:0009 651	1 gene	1(45)	7(29095)	0.010778	0.0314	TP53
cytoplasmic sequestering of transcription factor (BP)	GO:0042 994	1 gene	1(45)	7(29095)	0.010778	0.0314	MDFI
positive regulation of interleukin-12 biosynthetic process (BP)	GO:0045 084	1 gene	1(45)	7(29095)	0.010778	0.0314	RELA
positive regulation of SMAD protein nuclear translocation (BP)	GO:0060 391	1 gene	1(45)	7(29095)	0.010778	0.0314	RBPMS
positive regulation of transcription from RNA polymerase II promoter (BP)	GO:0045 944	3 genes	3(45)	291(290 95)	0.010308	0.0328	ZNF462,RELA,TP53
cellular component movement (BP)	GO:0006 928	2 genes	2(45)	101(290 95)	0.010719	0.0337	VIM,HSPB1
determination of adult lifespan (BP)	GO:0008 340	1 gene	1(45)	8(29095)	0.012308	0.0343	HTT
activation of caspase activity by cytochrome c (BP)	GO:0008 635	1 gene	1(45)	8(29095)	0.012308	0.0343	TP53
response to UV-B (BP)	GO:0010 224	1 gene	1(45)	8(29095)	0.012308	0.0343	RELA
positive regulation of chondrocyte differentiation (BP)	GO:0032 332	1 gene	1(45)	8(29095)	0.012308	0.0343	RELA
urea cycle (BP)	GO:0000 050	1 gene	1(45)	9(29095)	0.013836	0.0346	HTT
glutamate metabolic process (BP)	GO:0006 536	1 gene	1(45)	9(29095)	0.013836	0.0346	GLUD2
DNA damage response, signal transduction by p53 class mediator resulting in transcription of p21 class mediator (BP)	GO:0006 978	1 gene	1(45)	9(29095)	0.013836	0.0346	TP53
ER overload response (BP)	GO:0006 983	1 gene	1(45)	9(29095)	0.013836	0.0346	TP53
ATP hydrolysis coupled proton transport (BP)	GO:0015 991	1 gene	1(45)	9(29095)	0.013836	0.0346	ATP6V0A4
negative regulation of protein catabolic process (BP)	GO:0042 177	1 gene	1(45)	9(29095)	0.013836	0.0346	RELA
muscle cell differentiation (BP)	GO:0042 692	1 gene	1(45)	9(29095)	0.013836	0.0346	QKI
response to arsenic (BP)	GO:0046 685	1 gene	1(45)	9(29095)	0.013836	0.0346	SRRT
vesicle transport along microtubule (BP)	GO:0047 496	1 gene	1(45)	9(29095)	0.013836	0.0346	HTT
chaperone mediated protein folding requiring cofactor (BP)	GO:0051 085	1 gene	1(45)	9(29095)	0.013836	0.0346	HSPA8
organ morphogenesis (BP)	GO:0009 887	2 genes	2(45)	120(290 95)	0.014877	0.0368	RELA,HTT
regulation of Wnt receptor signaling pathway (BP)	GO:0030 111	1gene	1(45)	10(2909 5)	0.015362	0.0369	MDFI
COPI coating of Golgi vesicle (BP)	GO:0048 205	1 gene	1(45)	10(2909 5)	0.015362	0.0369	ARCN1

regulation of mitochondrial membrane potential (BP)	GO:0051881	1 gene	1(45)	10(29095)	0.015362	0.0369	HTT
endoplasmic reticulum organization (BP)	GO:0007029	1 gene	1(45)	11(29095)	0.016885	0.039	HTT
membrane protein intracellular domain proteolysis (BP)	GO:0031293	1 gene	1(45)	11(29095)	0.016885	0.039	RELA
negative regulation of tumor necrosis factor production (BP)	GO:0032720	1 gene	1(45)	11(29095)	0.016885	0.039	HSF1
protein tetramerization (BP)	GO:0051262	1 gene	1(45)	11(29095)	0.016885	0.039	TP53
regulation of mitosis (BP)	GO:0007088	1 gene	1(45)	12(29095)	0.018406	0.0406	KIF20B
rRNA transcription (BP)	GO:0009303	1 gene	1(45)	12(29095)	0.018406	0.0406	TP53
hormone metabolic process (BP)	GO:0042445	1 gene	1(45)	12(29095)	0.018406	0.0406	HTT
eye photoreceptor cell development (BP)	GO:0042462	1 gene	1(45)	12(29095)	0.018406	0.0406	CEP290
respiratory burst (BP)	GO:0045730	1 gene	1(45)	12(29095)	0.018406	0.0406	PGAM1
hair follicle development (BP)	GO:0001942	1 gene	1(45)	13(29095)	0.019925	0.0424	RELA
associative learning (BP)	GO:0008306	1 gene	1(45)	13(29095)	0.019925	0.0424	HTT
cortical actin cytoskeleton organization (BP)	GO:0030866	1 gene	1(45)	13(29095)	0.019925	0.0424	CALR
positive regulation of phagocytosis (BP)	GO:0050766	1 gene	1(45)	13(29095)	0.019925	0.0424	CALR
senescence (BP)	GO:0010149	1 gene	1(45)	14(29095)	0.021442	0.0431	CALR
hindbrain development (BP)	GO:0030902	1(gene	1(45)	14(29095)	0.021442	0.0431	CEP290
T cell differentiation in the thymus (BP)	GO:0033077	1 gene	1(45)	14(29095)	0.021442	0.0431	TP53
DNA damage response, signal transduction by p53 class mediator resulting in induction of apoptosis (BP)	GO:0042771	1 gene	1(45)	14(29095)	0.021442	0.0431	TP53
response to morphine (BP)	GO:0043278	1 gene	1(45)	14(29095)	0.021442	0.0431	RELA
positive regulation of cell cycle (BP)	GO:0045787	1 gene	1(45)	14(29095)	0.021442	0.0431	CALR
chromosome organization (BP)	GO:0051276	1 gene	1(45)	14(29095)	0.021442	0.0431	TP53
protein export from nucleus (BP)	GO:0006611	1 gene	1(45)	15(29095)	0.022956	0.045	CALR
induction of apoptosis by intracellular signals (BP)	GO:0008629	1 gene	1(45)	15(29095)	0.022956	0.045	TP53
response to amino acid stimulus (BP)	GO:0043200	1 gene	1(45)	15(29095)	0.022956	0.045	RELA
response to inorganic substance (BP)	GO:0010035	1 gene	1(45)	16(29095)	0.024468	0.0465	RELA
positive regulation of pathway-restricted SMAD protein phosphorylation	GO:0010862	1 gene	1(45)	16(29095)	0.024468	0.0465	RBPMS

(BP)							
social behavior (BP)	GO:0035176	1 gene	1(45)	16(29095)	0.024468	0.0465	HTT
negative regulation of insulin receptor signaling pathway (BP)	GO:0046627	1 gene	1(45)	16(29095)	0.024468	0.0465	RELA
negative regulation of DNA replication (BP)	GO:0008156	1 gene	1(45)	17(29095)	0.025977	0.0482	TP53
negative regulation of translation (BP)	GO:0017148	1 gene	1(45)	17(29095)	0.025977	0.0482	CALR
positive regulation of neuron apoptosis (BP)	GO:0043525	1 gene	1(45)	17(29095)	0.025977	0.0482	TP53
protein import into nucleus, translocation (BP)	GO:0000060	1 gene	1(45)	18(29095)	0.027485	0.0499	TP53
regulation of pH (BP)	GO:0006885	1 gene	1(45)	18(29095)	0.027485	0.0499	ATP6V0A4
negative regulation of gene-specific transcription (BP)	GO:0032582	1 gene	1(45)	18(29095)	0.027485	0.0499	RELA

Supplementary Table S4C: Enrichment of KEGG pathway with HYPK-interacting proteins

**Annotations**	**Total number of proteins out of 29095 proteins**	**HYPK-Interacting Proteins out of 45 protein identified**	**Corrected hypergeometric p Value**
(KEGG) 00471:D-Glutamine and D-glutamate metabolism	4	GLUD1, GLUD2	0.000329159
(KEGG) 04612:Antigen processing and presentation	81	HSP90AB1, CALR, HSPA8, PSME3	0.000357836
(KEGG) 05215:Prostate cancer	89	RELA, HSP90AB1, TP53	0.00560475
(KEGG) 00910: Nitrogen metabolism	23	GLUD1, GLUD2	0.00681114
(KEGG) 04010: MAPK signaling pathway	265	RELA, HSPB1, TP53, HSPA8	0.00702551
(KEGG) 00250:Alanine, aspartate and glutamate metabolism	31	GLUD1, GLUD2	0.00828026
(KEGG) 00330:Arginine and proline metabolism	53	GLUD1, GLUD2	0.0205831
(KEGG) 04621: NOD-like receptor signaling pathway	61	RELA, HSP90AB1	0.0210941
(KEGG) 00010:Glycolysis / Gluconeogenesis	59	PGAM1, TPI1	0.0222313
(KEGG) 05120:Epithelial cell signaling in Helicobacter pylori infection	68	RELA, ATP6V0A4	0.0234708
(KEGG) 05220:Chronic myeloid leukemia	75	RELA, TP53	0.0236635
(KEGG) 05212:Pancreatic cancer	72	RELA, TP53	0.0238474
(KEGG) 04210:Apoptosis	87	RELA, TP53	0.0270246
(KEGG) 05222:Small cell lung cancer	84	RELA, TP53	0.0271993
(KEGG) 05200:Pathways in cancer	325	RELA, HSP90AB1, TP53	0.0434377
(KEGG) 04722:Neurotrophin signaling pathway	124	RELA, TP53	0.0464941

Supplementary Table S4D: Enrichment of specific 16 pathways in the Reactome database with 49 HYPK-interacting proteins

False discovery rate	Un-adjusted probability of seeing N or more genes in this Event by chance	Number of genes in your query which map to this Event	Total number of genes involved in this Event	Identifier of this Event	Name of this Event	Submitted identifiers mapping to this Event
-	1.64E-06	3	6	REACT_25267	Association of AUF1 with Translation and Heat Shock Proteins	HSPB1, HSPA8, HSPA1A
-	9.72E-06	3	10	REACT_25315	Ubiquitination of AUF1 (hnRNP D0)	HSPB1, HSPA8, HSPA1A
-	8.89E-05	4	54	REACT_25325	Destabilization of mRNA by AUF1 (hnRNP D0)	HSPB1, HSPA8, PSME3, HSPA1A
-	0.000542031	4	86	REACT_24994	Regulation of mRNA Stability by Proteins that Bind AU-rich Elements	HSPB1, HSPA8, PSME3, HSPA1A
-	0.001669908	4	116	REACT_20605	Metabolism of mRNA	HSPB1, HSPA8, PSME3, HSPA1A
-	0.002062672	3	57	REACT_723	Glucose metabolism	CALM1, PGAM1, TPI1
-	0.00359101	4	143	REACT_21257	Metabolism of RNA	HSPB1, HSPA8, PSME3, HSPA1A
-	0.004352608	2	22	REACT_1383	Glycolysis	PGAM1, TPI1
-	0.008546792	2	31	REACT_1520	Gluconeogenesis	PGAM1, TPI1
-	0.015009709	3	116	REACT_474	Metabolism of carbohydrates	CALM1, PGAM1, TPI1
-	0.022179905	2	51	REACT_20549	Autodegradation of the E3 ubiquitin ligase COP1	PSME3, TP53
-	0.022179905	2	51	REACT_17044	Muscle contraction	CALM1, VIM
-	0.023003856	2	52	REACT_309	Stabilization of p53	PSME3, TP53
-	0.025551	2	55	REACT_1625	p53-Dependent G1 DNA Damage Response	PSME3, TP53
-	0.025551	2	55	REACT_85	p53-Dependent G1/S DNA damage checkpoint	PSME3, TP53
-	0.028208563	2	58	REACT_2254	G1/S DNA Damage Checkpoints	PSME3, TP53
