# Supplementary material for: Identification of HYPK-Interacting Proteins Reveals Involvement of HYPK in Regulating Cell Growth, Cell Cycle, Unfolded Protein Response and Cell Death
Source: PLoS One. 2012 Dec 10;7(12):e51415. doi: 10.1371/journal.pone.0051415 (PMC3525516; doi:10.1371/journal.pone.0051415)
Supplement: Table S5 — HYPK-interacting partners: sub-cellular localization and expression in brain. (PDF) [file pone.0051415.s011.pdf]

Supplementary Table S5: **HYPK interacting partners: sub-cellular localization and expression in brain**

	PROTEIN	Localization	Expression in Brain		
			**neXtProt**	**BioMart**	**TiGER**
	HYPK	Cytoplasm	Not available*	Not available*	YES
1	HTT	Cytoplasm, Nucleus	YES	YES	YES
2	ARCN1	Golgi apparatus membrane, COPI-coated vesicle membrane	YES	YES	YES
3	EEF1A1	Cytoplasm, Nucleus	YES	YES	YES
4	HSPA8	Cytoplasm	UBIQUITOUS	YES	YES
5	LMNB2	Nucleus inner membrane, Nucleoplasmic side, Lipid-anchor	YES	YES	YES
6	LBR	Nucleus inner membrane	YES	YES	YES
7	TPI 1	Nucleus	YES	YES	YES
8	SDCCAG1	Nucleus	YES	Not available*	YES
9	LENG8	Not available*	YES	YES	YES
10	IKBIP	Endoplasmic reticulum membrane	YES	YES	Not available*
11	CALM1	Spindle pole, Cell membrane, Golgi apparatus	YES	YES	YES
12	CDH11	Cell membrane	Not available*	YES	YES
13	CALR	Endoplasmic reticulum lumen, Cytosol, Extracellular matrix, Cell surface, Sarcoplasmic reticulum lumen	Not available*	YES	YES
14	CEP290	Centrosome, Nucleus	YES	YES	YES
15	ZNF462	Nucleus	YES	YES	YES
16	ZNF100	Nucleus	Not available*	YES	YES
17	ZNF516	Nucleus	Not available*	Not available*	YES
18	KIF20B	Spindle, Nucleus	YES	NO**	Not available*
19	HSP90AB1	Cytoplasm	YES	YES	YES
20	MYOM3	Not available*	Not available*	Not available*	YES
21	PGAM1	Cytosol	Not available*	Not available*	YES
22	N4BP1	Nucleolus (PML body)	Not available*	YES	YES
23	ATP6V0A4	Apical cell membrane	NO**	Not available*	NO**
24	GLUD1	Mitochondrion matrix	YES	YES	YES
25	GLUD2	Mitochondrion matrix	YES	YES	YES
26	SRRT	Nucleoplasm, Cytoplasm	Not available*	YES	Not available*
27	PPP6R2	Cytoplasm	NO**	YES	Not available*
28	NME2	Cytoplasm, Nucleus	UBIQUITOUS	Not available*	Not available*
29	VIM	Cytoplasm, Cell	Not available*	YES	YES

		membrane, Golgi apparatus			
30	HSF1	Cytoplasm, Nucleus	YES	Not available	YES
31	HSPA1A	Cytoplasm	YES	YES	YES
32	HSPB1	Cytoplasm, Nucleus, Cell membrane, Spindle	NO**	YES	YES
33	MLF1	Cytoplasm, Nucleus	YES	YES	YES
34	MLF2	Cytoplasm, Nucleus	YES	YES	YES
35	DNAJB3	Not available*	Not available*	Not available*	YES
36	RELA	Nucleus, Cytoplasm	YES	YES	YES
37	TP53	ER, Nucleus (PML BODY), Cytoplasm	Not available*	YES	YES
38	NAA10	Cytoplasm, Nucleus	YES	Not available*	Not available*
39	NAA15	Cytoplasm, Nucleus	NO**	YES	Not available*
40	HSPA14	Cytoplasm	Not available*	YES	YES
41	DNAJC2	Nucleus, cytoplasm	YES	YES	Not available*
42	CHD3	Nucleus, Cytoplasm (centrosome)	Not available	YES	YES
43	GC	Secreted	NO**	NO**	NO**
44	MDFI	Nucleus, Cytoplasm	Not available*	YES	Not available*
45	PSME3	Nucleus, Cytoplasm	YES	YES	YES
46	QKI	Nucleus, Cytoplasm	YES	YES	YES
47	RBPMS	Nucleus, Cytoplasm	Not available*	YES	Not available*
48	RHOXF2	Nucleus	NO**	NO**	Not available*
49	TH1L	Nucleus	Not available*	YES	Not available*

‘*’ **Not available:** No information (regarding the sub-cellular localization/expression in brain) was retrieved about the gene/protein from the concerned database

‘**’ **No:** This gene/protein does not express in brain
