# Supplementary material for: Identification of HYPK-Interacting Proteins Reveals Involvement of HYPK in Regulating Cell Growth, Cell Cycle, Unfolded Protein Response and Cell Death
Source: PLoS One. 2012 Dec 10;7(12):e51415. doi: 10.1371/journal.pone.0051415 (PMC3525516; doi:10.1371/journal.pone.0051415)
Supplement: Table S6 — Mean values and standard deviations for growth curve calculation of Neuro2A and ST HdhQ7 / HdhQ7 cells with and without HYPK. (PDF) [file pone.0051415.s012.pdf]

Supplementary Table S6: **Mean values and standard deviations for growth curve calculation of Neuro2A and ST*Hdh*^{Q7}/*Hdh*^{Q7} cells with and without HYPK**

A:

	ST*Hdh*^{Q7}/*Hdh*^{Q7} U61+DsRed	ST*Hdh*^{Q7}/*Hdh*^{Q7} HYPK U61+DsRed	ST*Hdh*^{Q7}/*Hdh*^{Q7} HYPK U61+HYPK-DsRed	ST*Hdh*^{Q7}/*Hdh*^{Q7} HYPK U61+HSPA8-DsRed
0 hr	100000	100000	100000	100000
24 hr	163333±4507	116666±1937	143333±1875	170000±1421
48 hr	303333±6866	220000±2457	300000±2045	276666±3457
72 hr	590000±6383	276666±2078	596666±4199	670000±2218
96 hr	1066667±26826	430000±5931	1100000±7845	1133333±4126
120 hr	1666667±12458	880000±6432	1900000±6241	1800000±5641

B:

	Neuro2A U61+DsRed	Neuro2A HYPK U61+DsRed	Neuro2A HYPK U61+HYPK-DsRed	Neuro2A HYPK U61+HSPA8-DsRed
0 hr	100000	100000	100000	100000
24 hr	140000±3426	120000±3647	130000±4843	145000±5405
48 hr	250000±5021	180000±4201	240000±3498	260000±2178
72 hr	400000±5689	250000±9472	370000±4708	420000±4294
96 hr	750000±6457	450000±4986	720000±8762	780000±5486
